# Identification of KW-2449 as a dual inhibitor of ferroptosis and necroptosis reveals that autophagy is a targetable pathway for necroptosis inhibitors to prevent ferroptosis

**DOI:** 10.1038/s41419-024-07157-9

**Published:** 2024-10-21

**Authors:** Yaxing Zhao, Qingsong Wang, Jing Zhu, Jin Cai, Xiaona Feng, Qianqian Song, Hui Jiang, Wenqing Ren, Yuan He, Ping Wang, Du Feng, Jianqiang Yu, Yue Liu, Qihui Wu, Siriporn Jitkaew, Zhenyu Cai

**Affiliations:** 1 Tongji University Cancer Center, Shanghai Tenth People’s Hospital, School of Medicine, Tongji University, Shanghai, China; 2 College of Pharmacy, Ningxia Medical University, Yinchuan, Ningxia Hui Autonomous Region, Yinchuan, China; 3 Department of Biochemistry and Molecular Biology, School of Medicine, Tongji University, Shanghai, China; 4 Guangzhou Municipal and Guangdong Provincial Key Laboratory of Protein Modification and Degradation, School of Basic Medical Sciences, Guangzhou Medical University, Guangzhou, China; 5 Shanghai Fourth People’s Hospital, School of Medicine, Tongji University, Shanghai, China; 6 Center of Excellence for Cancer and Inflammation, Department of Clinical Chemistry, Faculty of Allied Health Sciences, Chulalongkorn University, Bangkok, Thailand; 7 State Key Laboratory of Cardiology and Medical Innovation Center, Shanghai East Hospital, School of Medicine, Tongji University, Shanghai, China

**Keywords:** Necroptosis, Macroautophagy, Kinases

## Abstract

Necroptosis and ferroptosis are two distinct forms of necrotic-like cell death in terms of their morphological features and regulatory mechanisms. These two types of cell death can coexist in disease and contribute to pathological processes. Inhibition of both necroptosis and ferroptosis has been shown to enhance therapeutic effects in treating complex necrosis-related diseases. However, targeting both necroptosis and ferroptosis by a single compound can be challenging, as these two forms of cell death involve distinct molecular pathways. In this study, we discovered that KW-2449, a previously described necroptosis inhibitor, also prevented ferroptosis both in vitro and in vivo. Mechanistically, KW-2449 inhibited ferroptosis by targeting the autophagy pathway. We further identified that KW-2449 functioned as a ULK1 (Unc-51-like kinase 1) inhibitor to block ULK1 kinase activity in autophagy. Remarkably, we found that Necrostatin-1, a classic necroptosis inhibitor that has been shown to prevent ferroptosis, also targets the autophagy pathway to suppress ferroptosis. This study provides the first understanding of how necroptosis inhibitors can prevent ferroptosis and suggests that autophagy is a targetable pathway for necroptosis inhibitors to prevent ferroptosis. Therefore, the identification and design of pharmaceutical molecules that target the autophagy pathway from necroptosis inhibitors is a promising strategy to develop dual inhibitors of necroptosis and ferroptosis in clinical application.

## Introduction

Regulated cell death (RCD) plays a critical role in various physiological processes such as development, tissue homeostasis, and immune response. Dysregulation of RCD pathways can contribute to the development and progression of various diseases [1]. There are several types of RCDs that have been identified, including apoptosis, necroptosis, pyroptosis, and ferroptosis [2, 3]. Accumulating evidence suggests that different forms of RCD can occur together to mediate pathology and targeting multiple forms of RCD could be more effective in treating certain diseases [4].

Necroptosis and ferroptosis are two distinct forms of RCD that share several morphological characteristics with necrosis, such as cell swelling and plasma membrane rupture. Necroptosis is initiated by signals from death receptors of the tumor necrosis factor (TNF) superfamily, Toll-like receptors, interferon receptors, or Z-DNA binding protein 1 [5]. It is characterized by the activation of receptor-interacting protein kinase 1 and 3 (RIPK1 and 3) and phosphorylation of mixed lineage kinase domain-like protein (MLKL), leading to plasma membrane rupture and release of intracellular contents [6]. Distinct from necroptosis, ferroptosis is characterized by the accumulation of lipid peroxides and iron-dependent reactive oxygen species (ROS) in cells [7]. Ferroptosis can be triggered by the depletion of glutathione, a major intracellular antioxidant, or the inhibition of key enzymes involved in reducing lipid peroxides, such as glutathione peroxidase 4 (GPX4) [8]. This leads to the accumulation of lipid peroxides, which cause oxidative damage to cell membranes and ultimately result in cell death [9]. Recently, ferroptosis has been recognized as a form of autophagy-dependent cell death [10, 11]. Several types of selective autophagy, including ferritinophagy, lipophagy, clockophagy, and chaperone-mediated autophagy (CMA), have been shown to promote ferroptosis by inducing oxidative damage [11–15].

Necroptosis and ferroptosis can intersect or coexist in disease and contribute to pathological processes. For example, during ischemia-reperfusion-induced injury, ischemia can trigger necroptosis, while reperfusion promotes ferroptosis by inducing ROS generation and lipid peroxidation [16]. Neurodegenerative diseases, such as Alzheimer’s disease and Parkinson’s disease, often involve multiple mechanisms of cell death. In neurodegenerative conditions associated with inflammation and oxidative stress, both necroptosis and ferroptosis can contribute to neuronal cell death and disease progression [17]. In acute kidney injury (AKI), necroptosis and ferroptosis have been implicated in the pathogenesis of renal damage [18]. Inhibition of either necroptosis or ferroptosis has shown protective effects against AKI in preclinical studies [19]. Therefore, combining inhibitors of necroptosis and ferroptosis provides synergistic effects and better disease management in these conditions [20, 21].

Targeting both necroptosis and ferroptosis by a single compound can be challenging, as these two forms of RCD involve distinct molecular pathways. Currently, there are no specific dual inhibitors that exclusively target both necroptosis and ferroptosis. However, several necroptosis inhibitors have been identified that can modulate ferroptosis to some extent. For example, necroptosis inhibitor necrostatin-1 (Nec-1) and its derivative Nec-1f have been shown to suppress ferroptosis at relatively high concentrations [22–24]. Additionally, a derivative from a plant natural product named nigratine was recently identified to inhibit both necroptosis and ferroptosis [25]. However, the concentrations of these necroptosis inhibitors that effectively inhibit ferroptosis are relatively high. Furthermore, the action mechanism of Nec-1 or nigratine to inhibit ferroptosis is currently unknown. Therefore, identification of the molecular target(s) of these necroptosis inhibitors in ferroptosis is a prerequisite for improving their efficacy on ferroptosis-related phenotypes, which is critical to developing novel specific dual inhibitors of necroptosis and ferroptosis for potential clinical use.

In the present study, we discovered KW-2449, a previously described necroptosis inhibitor that targets RIPK1 activity, inhibited ferroptosis both in vitro and in vivo. Mechanistically, KW-2449 inhibited ferroptosis by targeting the autophagy pathway. We further identified KW-2449 as a ULK1 (Unc-51-like kinase 1) inhibitor to block ULK1 kinase activity in autophagy. Remarkably, we found Nec-1, a classic necroptosis inhibitor that has been reported to suppress ferroptosis, also targets the autophagy pathway to prevent ferroptosis. These findings suggest that the identification and design of pharmaceutical molecules that target the autophagy pathway from necroptosis inhibitors is a promising strategy to develop dual inhibitors of necroptosis and ferroptosis in clinical application.

## Materials and methods

### Chemicals

KW-2449 (Cat#HY-10339), Cisplatin (Cat#HY-17394), Erastin (Cat#HY-15763), RSL3 (Cat#HY-100218A), iFSP1 (Cat#HY-136057), Ferrostatin-1 (Fer-1; Cat#HY-100579), Ammonium iron(III) citrate (FAC; Cat#HY-B1645), Bafilomycin A1 (BafA1; Cat#HY-100558), Necrostatin-1 (Nec-1; Cat#HY-15760), PF-03814735 (Cat#HY-14574), GNF-7 (Cat#HY-10943), XST-14 (Cat# HY-137506), Smac mimetic (SM-164, Cat#HY-15989), Z-VAD-FMK (Cat#HY-16658B), H2DCFDA (Cat#HY-D0940), BODIPY 581/591 C11 (Cat#HY-D1301) and DPPH (Cat#HY-112053) were purchased from MedChemExpress (Monmouth Junction, USA). FLT3 inhibitor III (Cat#GC43676) was purchased from GlpBio (Montclair, USA). Iron (II) sulfate heptahydrate (FeSO_4_.7H_2_O; Cat#A600461) was purchased from Sangon Biotech (Shanghai, China). Liperfluo(Cat#L248), FerroOrange (Cat#F374), and Lipi-Green (Cat#LD02) were purchased from DOJINDO laboratories (Kumamoto, Japan). Recombinant mouse TNF-α (Cat#410-MT-025/CF) were purchased from the R&D System (Minneapolis, MN).

### Antibodies

The following antibodies were from commercial sources: anti-4 Hydroxynonenal (Abcam, USA, Cat#ab46545, 1:300 for Immunohistochemical); anti-LC3 (CST, USA, Cat#12741, 1:1000); anti-NCOA4 (Santa Cruz, USA, Cat#sc-373739, 1:1000). anti-FTH1 (HUABIO, China, Cat# ET1610-78, 1:1000). anti-phospho-mTOR (CST, USA, Cat#5536, 1:1000); anti-mTOR (CST, USA, Cat#2983, 1:1000); anti-phospho-ULK1 (CST, USA, Cat#14202, 1:1000); anti-ULK1 (CST, USA, Cat#8054, 1:1000); anti-phosphor-ATG14 (CST, USA, Cat#92340, 1:1000); anti-ATG14 (Proteintech, China, Cat#19491-1-AP, 1:1000); anti-Flag (ABclonal, China, Cat# AE004, 1:1000); anti-ARNTL (Proteintech, China, Cat#14268-1-AP, 1:1000); anti-GPX4 (ABclonal, China, Cat# A11243, 1:1000) and GAPDH (HUABIO, China, Cat# ET1601-4, 1:100,000).

### Plasmids and lentiviral particles

The mammalian cell expression plasmids of Flag-tagged mouse ATG5 (Cat#LPP-Mm01237-Lv101-100) and Flag-tagged mouse ULK1 (Cat#LPP-Mm05952-Lv101-100) were purchased from iGeneBio (Guangzhou, China). The mammalian expression plasmids of Flag-tagged human ULK1 (Cat#HG12031-NF) were purchased from SinoBiological (Beijing, China). Two short hairpin RNAs (shRNAs) targeting mouse ATG5 were purchased from Sigma-Aldrich. The shRNA against ATG5#1 (Cat#TRCN0000375819) and ATG5#2 (Cat#TRCN0000099432) corresponds to the 3′ untranslated region 1057–1077 and the coding sequence (CDS) region 93–113 relative to the first nucleotide of the start codon of mouse ATG5 (GenBank: NM_053069), respectively. The mammalian cell expression plasmid of BFP-tagged mouse FTH1 was generated by inserting the CDS of FTH1 (GenBank: NM_010239) into pLVX-IRES-hygromycin and then inserting the CDS of azure fluorescent protein (GenBank: AIR08544) at the C-terminal of FTH1. Lentivirus particles of pGMLV-GFP-hLC3-Puro (Cat#GM-0220LV04-1) were purchased from Genomeditech (Shanghai, China).

### Cell culture and transfection

All cell lines used in this study were obtained from the American Type Culture Collection (ATCC, https://www.atcc.org/). HT1080 cells were maintained in MEN media. H1299 cells were maintained in RPMI-1640 media. Other cells were maintained in DMEM media. All media were supplemented with 10% fetal bovine serum, 2 mM of L‐glutamine, and 100 U/ml of penicillin/streptomycin. Cells were cultured in a 37 °C incubator with a humidified atmosphere of 5% CO_2_. Paired wild-type (WT) and RIPK1^−^^/^^−^ MEF cells were kindly provided by Prof. Haibing Zhang (Chinese Academy of Sciences, China). Paired WT and ULK1^−^^/^^−^ MEF cells were kindly provided by Prof. Du Feng (Guangzhou Medical University, China).

The plasmids were transfected with Lipo293F™ transfection reagent (Beyotime, China) according to the manufacturer’s protocol. After 24 h, the cell lysates were analyzed by immunoblotting.

### Cell treatment, cell death, and cell viability assays

Cells were treated with cisplatin (50 μM) for the indicated time to induce cell death. Ferroptosis was induced by treatment with RSL3 (10 μM) or Erastin (10 μM) for the indicated time, respectively. Necroptosis was induced by pre-treatment with z-VAD-fmk (20 μM) and Smac mimetic (10 nM) for 30 min followed by stimulation with TNF-α (20 ng/mL) (TSZ) for 12 h. KW-2449 (1 μM) was pre-treated for 30 min before the cells were exposed to one of the above stimuli. If there are any differences, a detailed description will be given in the figure legend. Cell death was examined by propidium iodide (PI) staining. Briefly, cells were trypsinized, collected by centrifugation, washed once with PBS buffer, and then resuspended in PBS containing 5 μg/mL of PI. A total of 1 × 10^4^ events was counted by flow cytometry using a BD FACS Aria II (BD Biosciences, USA). The proportions of PI‐positive cells were quantified with FlowJo™ Software (BD Biosciences, USA). Cell viability was examined by using the CellTiter-Lumi™ Assay kit (Beyotime, China). Luminescence was recorded with a Tecan Spark microplate reader (Tecan Instruments, Switzerland).

### Lipid peroxidation assay

A total of 2 × 10^5^ cells were seeded in a six-well culture plate and incubated at 37 °C for 16 h. On the next day, cells were pre-treated with KW-2449 (1 μM) for 30 min and then treated with cisplatin (50 μM) for 12 h or RSL3 (10 μM) for 2 h to induce ferroptosis. Then, BODIPY 581/591 (5 μM) was added to cells and incubated at 37 °C for 30 min. Cells were washed with PBS three times. At least 1 × 10^4^ cells were collected for flow cytometry analysis with FL1 channel and analyzed by FlowJo^TM^ Software.

### Total reactive oxygen species (ROS) assay

A total of 2 × 10^5^ MEF cells were seeded in a six-well culture plate and incubated at 37 °C for 16 h. On the next day, cells were pre-treated with KW-2449 (1 μM) for 30 min and then treated with RSL3 (10 μM) for 2 h or H_2_O_2_ (5 mM) for 15 min or rotenone (5 μM) for 16 h. Cells were then incubated with H2DCFDA (10 μM) at 37 °C for 30 min and washed three times with PBS to remove unbound probes. Five representative images of the cells were acquired in each sample with three independent experiments using the fluorescence microscope. The MFI of cells was evaluated by cytometry analysis with FL1 channel and analyzed by FlowJo^TM^ Software.

### DPPH free-radical scavenging assay

DPPH free-radical scavenging assays were performed as described previously [24]. Briefly, DPPH solution (0.2 mM) in methanol was added to each of the 96-well plates (100 μL per well). Ferrostatin-1, used as a standard antioxidant, was added to positive control wells with a final concentration of 5 μM. KW-2449 was added to each well at indicated concentrations. Plates were mixed, covered, and incubated in the dark at room temperature for 10 min and then the absorbance was measured at 517 nm with a Tecan Spark microplate reader. All the experiments were performed in triplicate, and the scavenging ratio (%) of KW-2449 or Fer-1 was calculated as (Ac - Ai)/(Ac-An) × 100%, where Ai is the absorbance of KW-2449 or Fer-1 plus DPPH; An is the absorbance of KW-2449 or Fer-1 plus methanol and Ac is the absorbance of methanol plus DPPH.

### Intracellular ferrous iron measurement

A total of 5 × 10^4^ MEF cells were seeded in 35 mm glass bottom culture dishes and incubated at 37 °C for 24 h. On the next day, cells were pre-treated with KW-2449 (1 μM) for 30 min and then treated with RSL3 (10 μM) for 1 h. Cells were washed three times with HBSS, stained with FerroOrange (1 μM) in HBSS, and imaged immediately. Treatments were staggered to ensure precise staining duration. Five representative images were captured in each sample with three independent experiments for each condition under identical exposure times with a laser scanning confocal microscopy system (Nikon, Japan) using a 63×/1.4 DIC Plan-Apochromat oil immersion objective. To quantify levels of labile Fe^2+^, each image was divided into RGB elements, and only the red component was used for the analysis using the ImageJ software (NIH, USA). A constant threshold was selected to eliminate errors caused by manually selecting thresholds for different images. The MFI of intracellular Fe^2+^ was calculated by limiting it to the threshold. The results shown are means ± SD from three independent experiments.

### Live cell staining of lipid droplets (LDs)

A total of 2 × 10^5^ MEF cells were seeded in 35 mm glass bottom culture dishes and incubated at 37 °C for 24 h. On the next day, cells were treated with the above experimental protocol, washed three times by FBS-free DMEM media, and incubated at 37 °C in a CO_2_ incubator for 30 min in the presence of 100 nM Lipi-Green and 10 µg/mL Hoechst 33342. Five representative images were captured in each sample with three independent experiments by laser scanning confocal microscopy. LDs indicated by fluorescence aggregation, were counted manually. The number of LDs per cell was determined by dividing the total number of spots by the number of nuclei in each field. The results shown are means ± SD from three independent experiments.

### Lentiviral production and generation of stable cells

HEK293T cells were seeded in a 6-well plate and co-transfected with 900 ng psPAX2, 300 ng pMD2.G, and 1.2 µg of transfer plasmid (Flag-tagged ULK1 or ATG5, FTH1-tagged-BFP or shRNA-ATG5) per well by using Lipo293F™ transfection reagent. Viral supernatant was harvested at 48 h after transfection, filtered through a 0.45 µm syringe filter, and frozen at −80 °C. Targeted cells were seeded in a 6-well plate with medium density, and infected with 2 mL of filtered viral supernatant containing polybrene at a final concentration of 10 µg/ml for 24 h. After 24 h of infection, the cells were selected with 2 µg/mL puromycin or 10 µg/mL hygromycin B or 400 µg/mL G418 selective antibiotic.

### Generation of stable cells with GFP-LC3 expression and autophagy induction

MEF cells were infected with GFP-LC3-puro lentivirus and then selected with 2 µg/ml puromycin to generate a stable cell line with GFP-LC3 expression. A total of 5 × 10^4^ MEF cells with GFP-LC3 expression were seeded in 35 mm glass bottom culture dishes and incubated at 37 °C for 24 h. On the next day, cells were pre-treated with KW-2449 (1 μM) or Necrostatin-1 (10 μM) for 30 min and then treated with RSL3 (10 μM) or amino acid-deprived DMEM media for 1 h. Five representative images were captured in each sample with three independent experiments by laser scanning confocal microscopy system. GFP-expressing puncta, which is indicated by fluorescence aggregation, were counted manually. The number of LC3 puncta per cell was determined by dividing the total number of puncta by the number of cells in each field. The results shown are means ± SD from three independent experiments.

MEF cells with stably expressing LC3 were infected with BFP-tagged- FTH1 lentivirus and then selected with 10 µg/ml hygromycin B to generate a stable cell line with GFP-LC3 and FTH1-BFP expression. A total of 5 × 10^4^ transfected MEF cells were seeded in 35 mm glass bottom culture dishes and incubated at 37 °C for 24 h. On the next day, cells were pre-treated with KW-2449 (1 μM) for 30 min and then treated with RSL3 (10 μM) for 1 h. Five representative images were captured in each sample with three independent experiments by laser scanning confocal microscopy system. The colocalization of GFP and BFP were merged by laser confocal and counted manually. The number of colocalization puncta per cell was determined by dividing the total number of colocalization puncta by the number of cells in each field. Histograms on the gray value of fluorescence intensity of GFP and BFP channels were analyzed by ImageJ software (NIH, USA). The results shown are means ± SD from three independent experiments.

### Quantitative RT-PCR

RNA extraction using TRIzol reagent (ThermoFisher, USA) along with a chloroform extraction method. For gene expression analysis, total RNA was reverse-transcribed into cDNA using a Strand cDNA Synthesis kit (Transgen, China Cat#AH321-01). Levels of mRNA were measured by real-time PCR using PerfectStart® Green qPCR SuperMix (Universal Passive Reference Dye) (Transgen, China Cat#AQ602-01) in the QuantStudio 7 Flex Real-Time PCR system (ThermoFisher, USA). The ratio for the mRNA was normalized to GAPDH. The primer sequence is as follows:

FLT3 sense: 5′ - GAGCGACTCCAGCTACGTC -3′,

FLT3 antisense:5′ - ACCCAGTGAAAATATCTCCCAGA -3′;

Aurora sense: 5′ - CTGGATGCTGCAAACGGATAG -3′,

Aurora antisense:5′ - CGAAGGGAACAGTGGTCTTAACA -3′;

ABL sense: 5′ - CTCCAAGGAAAACCTTCTTGCT -3′,

ABL antisense:5′ - GCTGAGAGTGTTATCTCCACTGG -3′;

LAMP1 sense: 5′ - CAGCACTCTTTGAGGTGAAAAAC -3′,

LAMP1 antisense:5′ - ACGATCTGAGAACCATTCGCA -3′;

CTSB sense: 5′ - GCTTCCGGTCTTTGACAACCT -3′,

CTSB antisense:5′ - CACCAAGCATTAGTTCTCCTCC -3′;

FTH1 sense: 5′ - CAAGTGCGCCAGAACTACCAC -3′,

FTH1 antisense:5′ - GCCACATCATCTCGGTCAAAA -3′;

EGLN2 sense:5′ - AGTCCTTGGAGTCTAGCCGAG -3′,

EGLN2 antisense: 5′ - TGGCAGTGGTCGTAGTAGCAG -3′;

GAPDH sense: 5′ - GTTGTCTCCTGCGACTTCA -3′,

GAPDH antisense: 5′ - GGTGGTCCAGGGTTTCTTA -3′.

### siRNA-mediated gene knockdown

Dicer-substrate siRNAs were purchased from SYNBIO TECHNOLOGIES. All siRNAs were suspended at a concentration of ~10 μM using nuclease-free water. The sense strand of Dicer-substrate siRNAs used are as follows:

siRNA-Ctrl, UUCUCCGAACGUGUCACGUdTdT;

FLT3, GGUGUCGAGCAGUACUCUAAATT;

Aurora, CCUCAUUUCAAGACUGUUAAATT;

ABL, CUCCGGGUCUUGGGUUAUAAUTT.

For colocalization analysis, a total of 5 × 10^4^ MEF cells with stably expressing GFP-LC3 and FTH1-BFP were seeded in 35 mm glass bottom culture dishes and incubated at 37 °C for 24 h. On the next day, cells were transfected with individual siRNA for 48 h according to the manufacturer’s protocol, then treated with DMSO or RSL3 (10 μM) for 1 h. Five representative images were captured by laser scanning confocal microscopy system. The colocalization of GFP and BFP were merged by laser confocal and counted manually. For cell death assay, MEF cells were transfected with individual siRNA as mentioned above, and treated with DMSO or RSL3 (10 μM) for 6 h. Cell death was examined by propidium iodide (PI) staining. The results shown are means ± SD from three independent experiments.

### RNA-seq analysis

MEF cells were treated with DMSO control, RSL3 (10 μM), KW-2449 (1 μM) or

RSL3 plus KW-2449 pre-treatment for 2 h. The total RNA was extracted by using the TRIzol reagent (ThermoFisher, USA) and then employed for Illumina HiSeq library construction and sequencing according to the manufacturer’s instructions (Illumina, USA). The differentially expressed genes (DEGs) were identified by DESeq with the screening condition as follows: significant *p*-value < 0.05. A total of 221 AAGs from the Human Autophagy Database (http://www.autophagy.lu/index.html) were obtained. Based on 221 human AAGs, we identified a total of 217 mouse genes homologous to human AAGs by gene homology comparison. The overlapped genes between DEGs and mouse AAGs were analyzed by Venny 2.1 software (https://www.omicstudio.cn/tool). The heatmap was created by FindMarkers software (Seurat R package).

### Molecular docking

The 3D structure of KW-2449 was obtained from Pubchem (Compound CID: 11427553; https://pubchem.ncbi.nlm.nih.gov/). Human ULK1 (PDB ID: 4WNO) was selected as a modeling template and imported into the molecular docking software (Discovery Studio 2019, France). The solvent molecules were removed, and hydrogen atoms were added to the CHARMM force field. The coordinates of the ULK1 center were x = −9.027313, y = −40.802365, z = −14.319899 (Å = 7). Finally, KW-2449 was connected to the active cavity of ULK1 by means of CDOCKER.

### In vitro kinase assay

The inhibitory activity of KW-2449 on ULK1 kinase was determined by ULK1 Kinase Enzyme System kit (Promega, USA, Cat#V3521) and ADP-Glo™ Kinase Assay (Promega, USA, Cat#V6930) according to the manufacturer’s instruction. Briefly, KW-2449 was tested in triplicate in an 11-dose EC_50_ mode with two-fold serial dilution and a starting dose of 1 μM. The following components were added to the wells of 384 low-volume plates and incubated at room temperature for 60 min: 1 μL DMSO or KW-2449, 1 µL of ULK1 kinase enzyme (10 ng/µL), 3 µL of substrate/ATP mix (20 μM). Then 5 µL of ADP-Glo™ Reagent was added and incubated at room temperature for 60 min. After incubating, 10 µL of Kinase Detection Reagent was added and incubated at room temperature for 30 min. Finally, luminescence (Integration time 1 s) was recorded and the EC_50_ of KW-2449 on ULK1 kinase activity was calculated.

### Immunoblotting

Cell lysates were prepared for immunoblotting analysis using RIPA buffer supplemented with protease/phosphatase inhibitors and PMSF (MCE, Shanghai, China). The RIPA buffer consisted of 10 mM Tris-HCl [pH 8.0], 1 mM EDTA, 0.5 mM EGTA, 140 mM NaCl, 0.1% SDS, 1% Triton X-100, 50 mM NaF, 40 mM glycerophosphate, and 0.1 mM sodium vanadate. Cell lysates were separated by SDS‐PAGE and analyzed by immunoblotting. The dilution of the antibodies used for Western blotting is 1:1000. The proteins were visualized by enhanced chemiluminescence according to the manufacturer’s instructions (Tanon, China).

### Animal experiment

All animal care and experimental procedures complied with the National Institutes of Health guidelines and were approved by the animal care and use committee of Tongji University. Animal studies are reported in compliance with the ARRIVE guidelines [26]. No samples or animals were excluded from the analysis in this study. Mice were bred and housed under SPF conditions in individually ventilated cages with a 12 h light/dark cycle and stable temperature (25 °C). All mice used in the experiments were between the age of 8–10 weeks. Meanwhile, no statistical method was used to predetermine mouse size. C57BL/6 mice were purchased from Gem Pharmatech Co., Ltd unless mentioned otherwise. MLKL^−^^/^^−^ mice with C57BL/6 background were kindly provided by Prof. Jiahuai Han (Xiamen University, China).

### Cisplatin-induced AKI

Cisplatin-induced AKI was performed as described previously [27]. Briefly, eight to ten-week-old male WT and MLKL^−^^/^^−^ mice were randomly divided into four groups: Saline (n = 6), Cisplatin (n = 6), KW-2449 (n = 6), Cisplatin plus KW-2449 (n = 6) in this study. KW-2449 was dissolved in 0.5% methylcellulose 400 solution and administered once daily via oral gavage at 10 mg/kg 1.5 h before the injection of cisplatin. Cisplatin was dissolved in 0.9% saline for intraperitoneal administration at a single dose of 25 mg/kg. After 72 h, the mice were sacrificed by CO_2_ inhalation and the kidneys were removed for further study.

### Iron overload-induced multiple organ dysfunction syndrome (MODS)

MODS was performed as described previously [28]. Briefly, the mice were randomly divided into four groups: Saline (n = 6), Iron (II) sulfate heptahydrate (n = 6), KW-2449 (n = 6), Iron (II) sulfate heptahydrate plus KW-2449 (n = 6). Iron (II) sulfate heptahydrate was dissolved in sterile saline with a final concentration of 50 mg/mL. KW-2449 was dissolved in 0.5% sodium carboxymethyl cellulose solution and administered every 12 h via oral gavage at 10 mg/kg. Male mice were intraperitoneally injected with iron (II) sulfate heptahydrate (500 mg/kg body weight) after the last oral administration of KW-2449 for 2 h. In the survival experiment, mice were continuously observed for 72 h and their survival proportions were recorded. In another experiment, after 4 h of Iron (II) sulfate heptahydrate treatment, around 200 μL blood samples were collected by facial vein to evaluate the serum levels of glutamic oxaloacetic transaminase (AST) and alanine transaminase (ALT). Following this, the mice were sacrificed using CO_2_ inhalation, and their livers were removed for further study.

### Immunohistochemical staining

Kidneys and livers were soaked in formalin for at least 24 h before wrapping it in paraffin. Paraffin blocks were sectioned and stained with the 4-HNE (1:300) antibody. The positive cell staining for 4-HNE was calculated by ImageJ software. The percentage of 4-HNE-positive cells was determined by dividing the total number of 4-HNE-positive cells by the number of cells per high-power field. The results shown are means ± SD from three independent experiments.

### In situ Liperfluo dye loading

In situ, Liperfluo dye loading in live Kidney tissue was conducted according to a previous study [29]. Briefly, kidneys were excised immediately after CO_2_ inhalation as described above and submerged rapidly in a 4 °C oxygenated Krebs solution for sectioning. The kidneys were sliced into 300-μm sections. Sections submerged in Krebs solution were stained with Liperfluo dye (5 μM) at 37 °C for 4 h, and washed with PBS three times. Then, kidney slices were labeled with DAPI for 15 min and fixed with 10% paraformaldehyde. Five representative images were captured by laser scanning confocal microscopy.

### Biochemistry assay of liver enzymes in serum and MDA in tissue homogenate

The serum in blood samples from mice was separated through centrifugation at 3000 rpm for 20 min. The levels of liver enzymes, including AST and ALT, were determined by utilizing the AST and ALT assay kits (Nanjing Jiancheng, China, Cat#C010-2-1; Cat#C009-2-1). The absorbance at 510 nm was measured using a Tecan Spark microplate reader (Tecan Instruments, Switzerland).

The kidney or liver tissue homogenate from mice was separated by centrifuging at 8000 rpm for 10 min. Homogenate supernatant was collected and used to detect the level of MDA by the MDA assay kits (Nanjing Jiancheng, China, Cat#A003-1-2). The absorbance at 532 nm was measured using a Tecan Spark microplate reader.

### Statistical analysis

The student’s t-test and one-way analysis of variance (ANOVA) were used for comparison among all different groups represented with the mean values ± standard errors. Log-rank (Mantel–Cox) test was performed for survival curve analysis using GraphPad Prism 8 (GraphPad Software, USA). All experiments were repeated at least three times with similar results. p < 0.05 was considered statistically significant.

## Results

### Necroptosis inhibitor KW-2449 suppresses cisplatin-induced ferroptosis

**Fig. 1:**
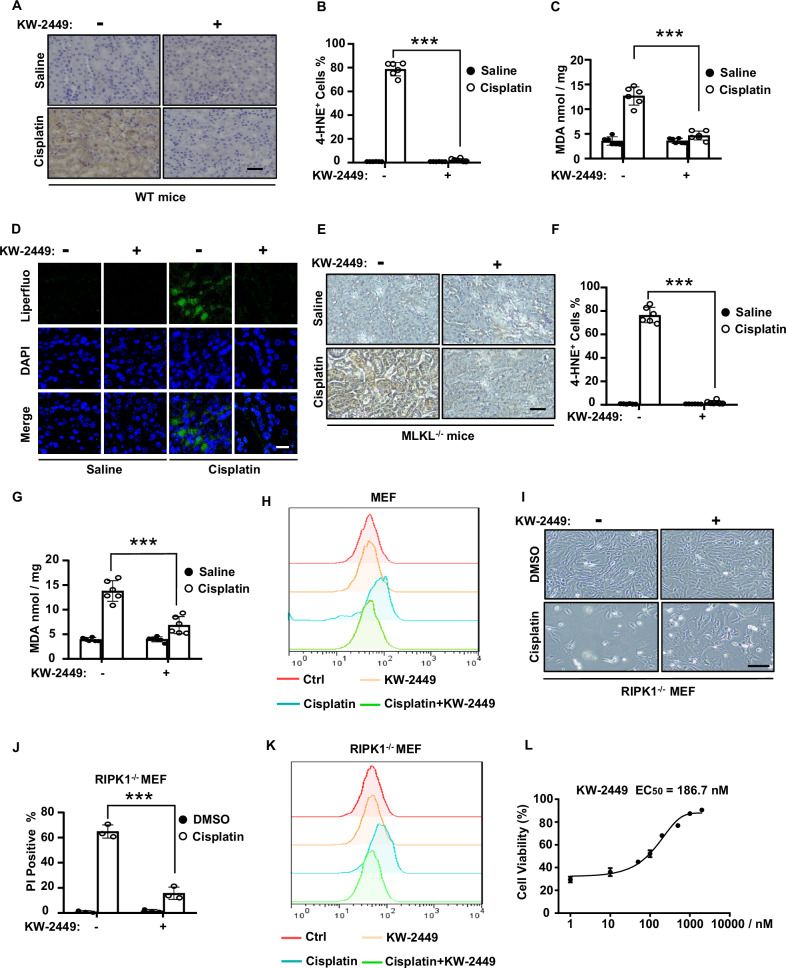
KW-2449 inhibits cisplatin-induced ferroptosis. **A** Representative images of immunohistochemical staining of 4-HNE in kidney tissues from wild-type (WT) mice (n = 6 for each group) treated with saline or cisplatin (25 mg/kg), with or without KW-2449 (±KW-2449, 10 mg/kg, i.g.). Scale bar: 50 μm. **B** The percentage of 4-HNE-positive cells in (**A**). **C** Quantification of MDA levels in renal homogenate from the mice in (**A**). **D** Representative images of Immunofluorescence staining of Liperfluo in kidney tissues from WT mice (n = 6 for each group) treated as in (**A**), Scale bar: 50 μm. **E** Representative images of immunohistochemical staining of 4-HNE in kidney tissues from MLKL-deficient mice (n = 6 for each group) treated as in (**A**), Scale bar: 50 μm. **F** Quantification of the percentage of 4-HNE-positive cells in (**E**). **G** Quantification of MDA levels in renal homogenate from the mice in (**E**). **H** MEF cells were pre-treated with DMSO or KW-2449 for 30 min followed by treatment with cisplatin for 12 h. Lipid peroxidation was then evaluated by flow cytometry using BODIPY dye. **I** RIPK1-deficient MEF cells were pre-treated with DMSO or KW-2449 for 30 min followed by treatment with cisplatin for 24 h. Representative images were shown. Scale bar: 50 μm. **J** Cell death was detected by flow cytometry after PI staining. **K** Lipid peroxidation was evaluated by flow cytometry using BODIPY dye. **L** RIPK1-deficient MEF cells were pre-treated with KW-2449 at indicated concentrations for 30 min and then followed by treatment with cisplatin for 24 h. Cell viability was analyzed by CellTiter-Lumi assay. Bar graphs represent the mean ± SD from three independent experiments. Statistical analysis was performed using a two-sided student’s t-test. ***p < 0.001.

We previously demonstrated that the multi-targeted kinase inhibitor KW-2449 was a necroptosis inhibitor by suppressing RIPK1 kinase activity [27]. Since necroptosis is involved in cisplatin-induced AKI [30], we further showed that KW-2449 prevented cisplatin-induced renal damage and inflammation in mice [27]. Besides necroptosis, ferroptosis also contributes to cisplatin-induced AKI and several ferroptosis inhibitors have been shown to alleviate cisplatin-induced nephrotoxicity [19–21]. We then asked whether KW-2449 had effects on ferroptosis in mice models of cisplatin-induced AKI. As lipid peroxidation is a hallmark of ferroptosis [7, 31], we examined two in vivo lipid peroxidation markers of ferroptosis [32], 4-hydroxynonenal (4-HNE) and malondialdehyde (MDA) in kidney tissues. We found that the levels of 4-HNE and MDA were both increased in the kidney tissue of cisplatin-treated mice, while these increases were significantly inhibited by KW-2449 (Fig. 1A–C). We further used Liperfluo, a specific fluorescent probe that is sensitive to lipid peroxidation [29, 33], to determine the levels of lipid peroxides in cisplatin-treated kidney tissues. By in situ loading of fluorescent probe Liperfluo in unfixed kidney slices, we found cisplatin greatly increased Liperfluo signals in kidney tissues, however, we could not detect the Liperfluo signals when KW-2449 was administrated in cisplatin-treated mice (Fig. 1D). Thus, these data suggest that necroptosis inhibitor KW-2449 prevents lipid peroxidation and ferroptosis in cisplatin-induced AKI. To elucidate whether the inhibitory effect of KW-2449 on necroptosis contributes to its activity in ferroptosis inhibition, we used MLKL-deficient mice to block cisplatin-induced necroptosis and found that loss of MLKL had no effect on cisplatin-induced production of 4-HNE and MDA (Fig. 1E–G), suggesting necroptosis and ferroptosis act independently in cisplatin-induced AKI. Furthermore, KW-2449 still inhibited cisplatin-induced production of 4-HNE and MDA in MLKL-deficient mice (Fig. 1E–G). Thus, these data indicate that KW-2449 inhibits ferroptosis in mice models of cisplatin-induced AKI, which is not dependent on its inhibitory activity in necroptosis.

As KW-2449 inhibits ferroptosis in cisplatin-induced AKI in vivo, we then examined whether it inhibited cisplatin-induced ferroptosis in cellular models. To examine whether ferroptosis is induced by cisplatin, we used a lipid peroxidation–sensitive dye BODIPY 581/591 C11 (BODIPY) to estimate the cellular levels of lipid peroxidation [34]. In mouse embryonic fibroblast cells (MEFs), we found cisplatin-induced lipid peroxidation, as measured by BODIPY dye, while the probe oxidation was inhibited by KW-2449 (Fig. 1H). These data suggest that cisplatin is able to induce ferroptosis in MEFs, which can be inhibited by KW-2449.

Since KW-2449 is a RIPK1 kinase inhibitor that suppresses RIPK1-mediated necroptosis [27], it may target RIPK1 to prevent cisplatin-induced ferroptosis. To test this possibility, we used RIPK1-deficient MEFs to block RIPK1-mediated necroptosis and treated these cells with cisplatin. Due to the blockage of necroptosis signaling pathway, cisplatin-induced cell death was attenuated in RIPK1-deficient MEFs (Fig. S1A). We found multiple ferroptosis inhibitors, including antioxidants Ferrostatin-1 (Fer-1), Trolox, and iron chelator deferoxamine (DFO), could inhibit cisplatin-induced cell death in these cells, suggesting ferroptosis is induced by cisplatin in RIPK1-deficient MEFs (Fig. S1B, C). Remarkably, KW-2449 protected these cells from cisplatin-induced cell death (Fig. 1I, J). By examining the levels of lipid peroxidation with BODIPY dye, we confirmed that cisplatin was able to induce ferroptosis in RIPK1-deficient MEFs, which can be suppressed by KW-2449 (Fig. 1K). Finally, the half maximal effective concentration (EC_50_) of KW-2449 for inhibiting cell death in this setting was measured to be 186.7 nM (Fig. 1L). Thus, these data suggest that KW-2449 inhibits ferroptosis, which is not dependent on its kinase inhibitory activity of RIPK1.

In addition to RIPK1 kinase, KW-2449 has a unique kinase inhibitory profile against FLT3, ABL, T315I-mutant ABL (ABL-T315I) kinases as well as aurora kinases [35]. We found the selective kinase inhibitors for FLT3, ABL or Aurora kinase did not inhibit RSL3-induced ferroptosis, respectively (Fig. S2A). We then knocked down these kinases by small interference RNA (siRNA), respectively (Fig. S2B), and found that siRNA-mediated knockdown of these genes has no effect on RSL3-induced ferroptosis (Fig. S2C, D). Thus, these data indicate that all known targets of KW-2449 do not contribute to its inhibitory effects in ferroptosis and KW-2449 must target other unidentified factor(s) to prevent ferroptosis. Taken together, our data suggest that KW-2449 inhibits two types of necrotic-like RCD, necroptosis, and ferroptosis, in cisplatin-induced cell death.

### KW-2449 inhibits cell death in multiple cellular models of ferroptosis

**Fig. 2:**
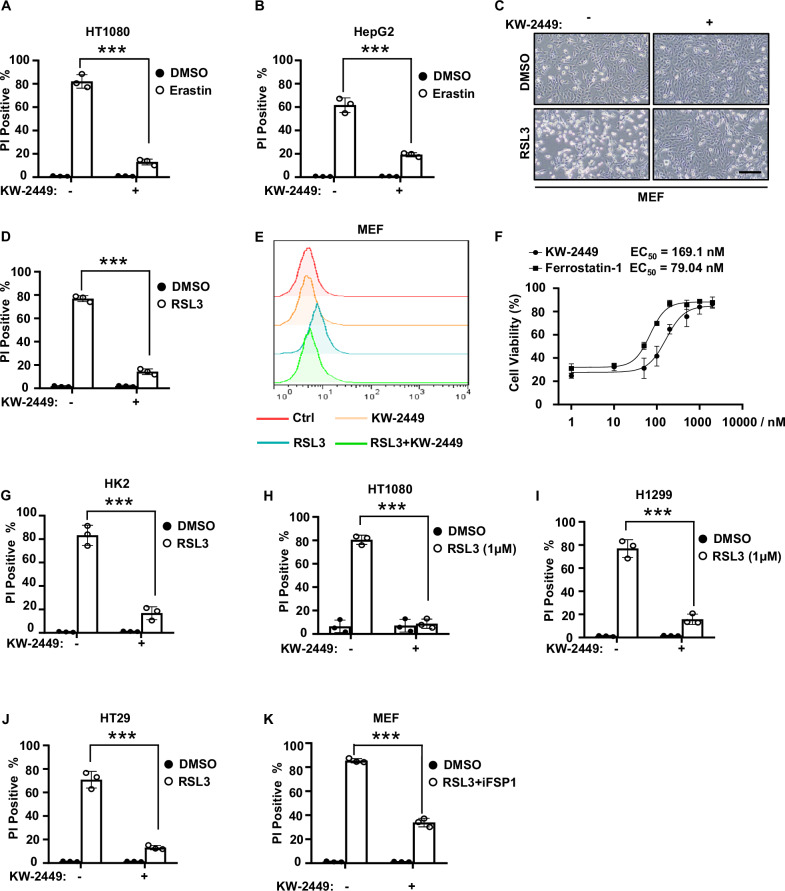
KW-2449 inhibits cell death in multiple cellular models of ferroptosis. **A** HT1080 or **B** HepG2 cells were pre-treated with DMSO or KW-2449 for 30 min followed by treatment with Erastin for 24 h. Cell death was detected by flow cytometry after PI staining. **C** MEF cells were pre-treated with DMSO or KW-2449 followed by treatment with RSL3 for 6 h. Representative images were shown. Scale bar: 50 μm. **D** Cell death of the MEF cells in (**C**) was detected by flow cytometry after PI staining. **E** Lipid peroxidation of the MEF cells in (**C**) was evaluated by flow cytometry using BODIPY dye. **F** MEF cells were pre-treated with KW-2449 or Fer-1 at indicated concentrations for 30 min followed by treatment with RSL3 for 6 h. Cell viability was analyzed by CellTiter-Lumi assay. **G** HK2, **H** HT1080, **I** H1299, or **J** HT29 cells were pre-treated with DMSO or KW-2449 for 30 min followed by treatment with RSL3 for 6 h. **K** MEF cells were pre-treated with DMSO or KW-2449 for 30 min followed by treatment with RSL3 plus iFSP1 for 4 h. Cell death was evaluated by flow cytometry after staining with PI. Bar graphs represent the mean ± SD from three independent experiments. Statistical analysis was performed using a two-sided student’s t-test. ***p < 0.001.

We then investigate the ability of KW-2449 to inhibit cell death in other classic cellular models of ferroptosis. Ferroptosis inducers (FINs) at least can be classified into three groups [8]: Class I FINs inhibit system xc^−^ such as Erastin; Class II FINs inhibit GPX4 such as RSL3; Class III FINs inhibit both GPX4 and CoQ_10_ pathway. As for Class I FIN-induced cell death, we found KW-2449 inhibited Erastin-induced ferroptosis in human fibrosarcoma HT1080 cells and liver cancer HepG2 cells (Fig. 2A, B). As for Class II FIN-induced cell death, we found KW-2449 inhibited RSL3-induced ferroptosis and lipid peroxidation in MEFs (Fig. 2C–E). And the EC_50_ of KW-2449 for inhibiting RSL3-induced ferroptosis was 169.1 nM, which was approximately 2-fold greater than the classical ferroptosis inhibitor Fer-1 (Fig. 2F). Furthermore, KW-2449 also prevented RSL3-induced ferroptosis in various human cell lines, including human renal proximal tubule HK2 cells, human fibrosarcoma HT1080 cells, human lung cancer H1299 cells and human colon cancer HT29 cells (Fig. 2G–J). As for Class III FIN-induced cell death, we found that KW-2449 inhibited both RSL3 and iFSP1-induced ferroptosis in MEFs (Fig. 2K). Thus, our data indicate that KW-2449 is a potent and effective ferroptosis inhibitor in multiple cellular models of ferroptosis.

### KW-2449 inhibits ROS production by preventing the accumulation of ferrous iron in ferroptosis

**Fig. 3:**
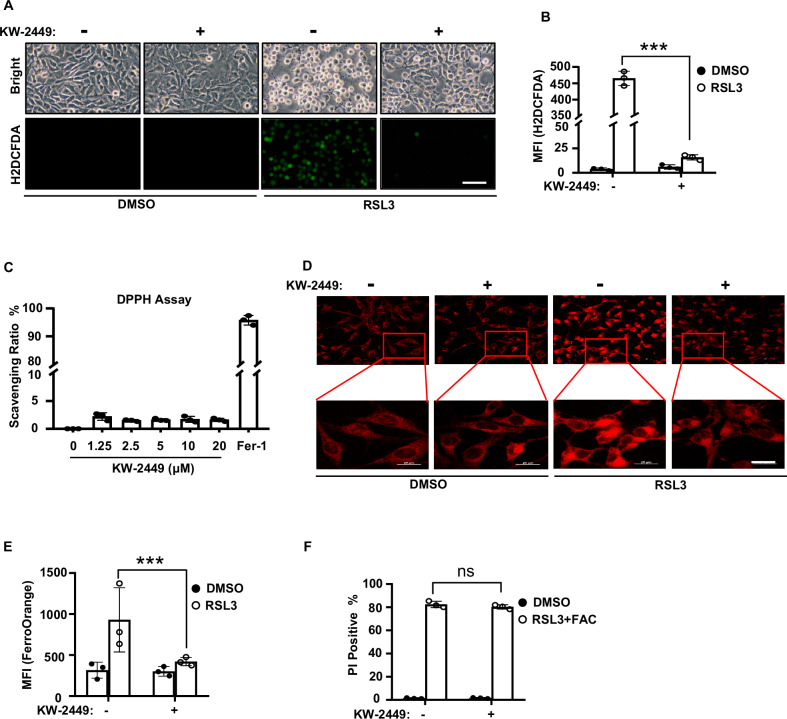
KW-2449 inhibits ROS production by preventing the accumulation of ferrous iron in ferroptosis. **A** MEF cells were pre-treated with DMSO or KW-2449 for 30 min followed by treatment with RSL3 for 2 h. Cells were stained with ROS fluorescent probe H2DCFDA and the representative images were shown. Scale bar, 40 µm. **B** The mean fluorescence intensity (MFI) of H2DCFDA from the cells in (**A**) was determined by flow cytometry. **C** The DPPH free-radical scavenging capacities of KW-2449 at indicated concentrations and Fer-1 (5 μM) were shown. **D** MEF cells were pre-treated with DMSO or KW-2449 for 30 min followed by treatment with RSL3 for 1 h. Cells were stained with a FerroOrange fluorescent probe to determine the concentrations of intracellular ferrous (Fe^2+^). The representative images were shown. Scale bar, 20 µm. **E** The MFI of FerroOrange from the cells in (**D**) was evaluated by ImageJ software (NIH, USA). **F** MEF cells were pre-treated with DMSO or KW-2449 for 30 min followed by treatment with RSL3 in combination with FAC (16 mM) for 4 h. Cell death was determined by flow cytometry after staining with PI. Bar graphs represent the mean ± SD. Statistical analysis was performed using a two-sided student’s t-test. The levels of significance were indicated by ***P < 0.001; ns not significant.

As ROS-induced lipid peroxidation plays a critical role in ferroptosis [7], we then examined whether KW-2449 inhibited ROS production in ferroptosis. By using a fluorescent ROS indicator H2DCFDA, we found that KW-2449 significantly inhibited ferroptosis-associated ROS production in MEFs (Fig. 3A, B), while it had no effects on hydrogen peroxide-induced ROS accumulation (Fig. S3A, B) or rotenone-induced mitochondrial ROS production (Fig. S3C, D). Furthermore, unlike the ferroptosis inhibitor Fer-1, KW-2449 did not display radical scavenging activity in cell-free 2,2-diphenyl-1-picrylhydrazyl (DPPH) assay over the concentration range showing blockade of ferroptosis (Fig. 3C). Thus, these data indicate that KW-2449 selectively inhibits ROS production in ferroptosis, which is not relative to radical scavenging activity.

Ferroptosis is a type of iron-dependent RCD and cellular iron is required for ROS accumulation [7]. In this scenario, excess Fe^2+^ can induce ferroptosis through reactive oxygen species generated by the Fenton reaction. We then measured the level of intracellular ferrous iron by FerroOrange staining. We found that RSL3 treatment led to the accumulation of ferrous (Fe^2+^) iron, while KW-2449 blocked the increase of ferrous iron in MEFs (Fig. 3D, E). This observation suggests that KW-2449 may block ferroptosis by decreasing the accumulation of ferrous iron in cells. To test this possibility, we then treated cells with ammonium citrate (FAC), an orally active iron supplement that can induce intracellular iron overload. We found that RSL3 plus FAC significantly induced cell death in MEFs, however, by increasing intracellular iron load with FAC, KW-2449 no more protected cells from RSL3 plus FAC-induced cell death (Fig. 3F). Thus, these data suggest that KW-2449 may inhibit the accumulation of ferrous iron to prevent ferroptosis.

### KW-2449 inhibits autophagy in ferroptosis

**Fig. 4:**
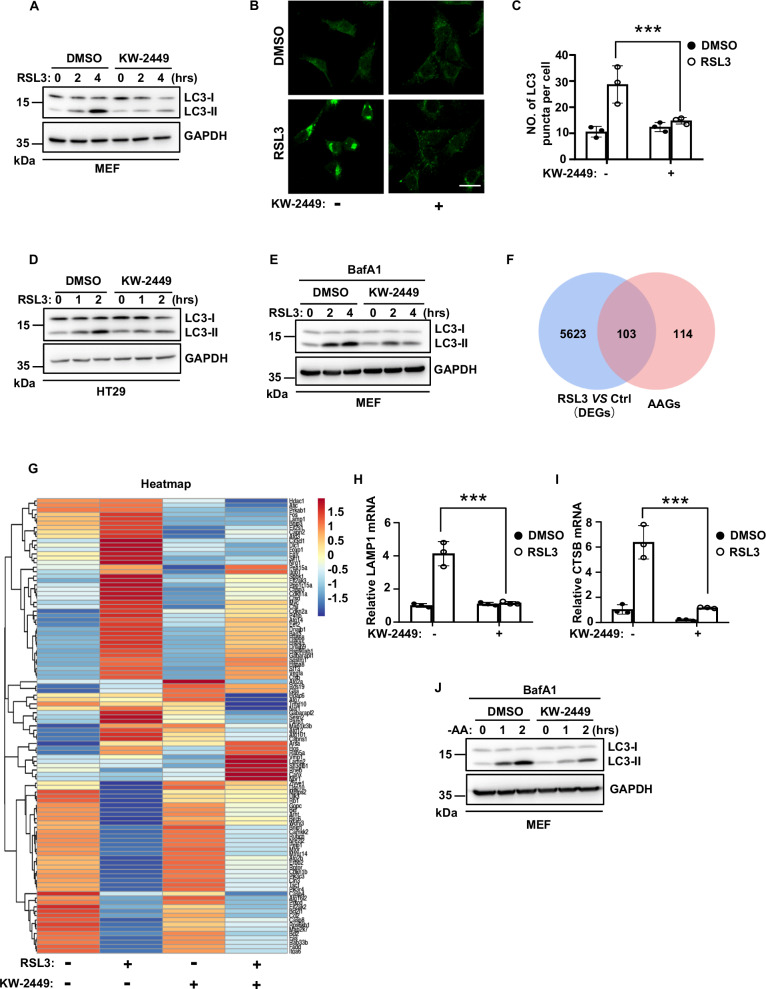
KW-2449 inhibits autophagy in ferroptosis. **A** MEF cells were pre-treated with DMSO or KW-2449 for 30 min followed by treatment with RSL3 for the indicated time. Cells were lysed and immunoblotted with LC3 antibody. **B** MEF cells stably expressed GFP-LC3 were pre-treated with DMSO or KW-2449 for 30 min followed by treatment with RSL3 for 1 h. Representative confocal images of the cells were shown. Scale bar, 50 µm. **C** Statistic analysis of LC3 puncta formation in MEF cells from (**B**). **D** HT29 cells were pre-treated with DMSO or KW-2449 for 30 min followed by treatment with RSL3 for the indicated time. Cells were lysed and immunoblotted with LC3 antibody. **E** MEF cells were pre-treated with DMSO or KW-2449 plus BafA1 (0.4 μM) for 30 min, followed by treatment with RSL3 for the indicated time. Cells were lysed and immunoblotted with the LC3 antibody. **F** MEF cells were treated with DMSO control, RSL3(10 μM), KW-2449 (1 μM), or RSL3 plus KW-2449 for 2 h. Venn diagram shows the overlap between 217 AAGs and 5726 genes (DEGs) differentially expressed between RSL3-treated and DMSO control cells (*p* < 0.05). **G** Heatmap shows 64 upregulated and 39 downregulated genes in RSL3-treated MEF cells from overlap analysis in (**F**). **H** MEF cells were pre-treated with DMSO or KW-2449 for 30 min followed by treatment with RSL3 for 2 h. Relative mRNA levels of LAMP1 and **I** CTSB were determined by RT-PCR. **J** MEF cells were pre-treated with DMSO or KW-2449 plus BafA1 for 30 min followed by starvation of amino acid for the indicated time. Cells were lysed and immunoblotted with LC3 antibody. Bar graphs represent the mean ± SD from three independent experiments. All Western data are representative of three independent experiments. Statistical analysis was performed using a two-sided student’s t-test. The levels of significance were indicated by ***P < 0.001.

The increase of intracellular ferrous iron in ferroptosis could be mediated by ferritinophagy, which is a selective type of autophagy that degrades iron storage protein ferritin to release labile iron in cells [8]. As ferroptosis-inducing conditions can trigger autophagic flux to promote ferroptotic cell death [11, 12], we first examined whether KW-2449 had an effect on autophagy during RSL3-induced ferroptosis. As shown in Fig. 4A, B, RSL3 induced a conversion of LC3-I to LC3-II and the formation of GFP-LC3 puncta, the hallmarks of autophagy response [36]. Remarkably, KW-2449 prevented RSL3-induced increase of LC3-II and clustering of GFP-LC3 puncta in MEFs (Fig. 4A–C). Similar results were also observed in human HT29 cells (Fig. 4D). As the accumulation of LC3-II may represent either an increase in autophagosome formation and/or a block of autophagic flux, we then treated cells with lysosomal inhibitor bafilomycin A1 (BafA1) to block lysosomal degradation of autophagosomes. Under this condition, we found RSL3 induced a significant increase in LC3-II expression, which could be inhibited by KW-2449 treatment, suggesting KW-2449 inhibits autophagosome formation in ferroptosis-associated autophagy (Fig. 4E). To gain more insights on the role of KW-2449 in ferroptosis-associated autophagy, we performed differential gene expression analysis from RNA-seq data in MEFs. We first obtained a total of 221 autophagy-associated genes (AAGs) from the Human Autophagy Database (http://www.autophagy.lu/index.html). Based on these 221 human AAGs, we identified 217 mouse genes homologous to human AAGs by gene homology comparison (Table S1). We then used these 217 genes as mouse AAGs and found that there were 103 mouse AAGs that differentially expressed between RSL3-treated and DMSO control cells (Fig. 4F). Among these 103 AAGs, we identified 64 genes were upregulated and 39 genes were downregulated (Fig. 4G and Table S2). Remarkably, we found the upregulated 64 AAGs in RSL3-treated cells were largely inhibited by KW-2449, while the downregulated 39 AAGs in RSL3-treated cells were largely upregulated by KW-2449 (Fig. 4G). Thus, these data suggest that KW-2449 has a global inhibitory effect on the transcriptional regulatory network of autophagy-related genes in ferroptosis. Our data therefore collectively indicate that KW-2449 is a bona fide inhibitor of RSL3-induced autophagy. Additionally, by using RT-RCR assay, we confirmed that the two AAGs identified by RNA-Seq, LAMP1, and CTSB, were both upregulated by RSL3 and downregulated by KW-2449 in RSL3-treated cells (Fig. 4H, I). Since LAMP1 and CTSB are required for the activation of autophagy [37, 38], this data further suggests that KW-2449 inhibits autophagic activity in ferroptosis. As the most typical trigger of autophagy is nutrient starvation, we then treated cells with amino acid (AA)-free medium to induce autophagy and found KW-2449 also inhibited the accumulation of LC3-II during AA deprivation in MEFs (Fig. 4J). Therefore, these data indicate that KW-2449 is an autophagy inhibitor to prevent autophagosome formation in ferroptosis.

### KW-2449 inhibits ferroptosis by targeting the autophagy pathway

**Fig. 5:**
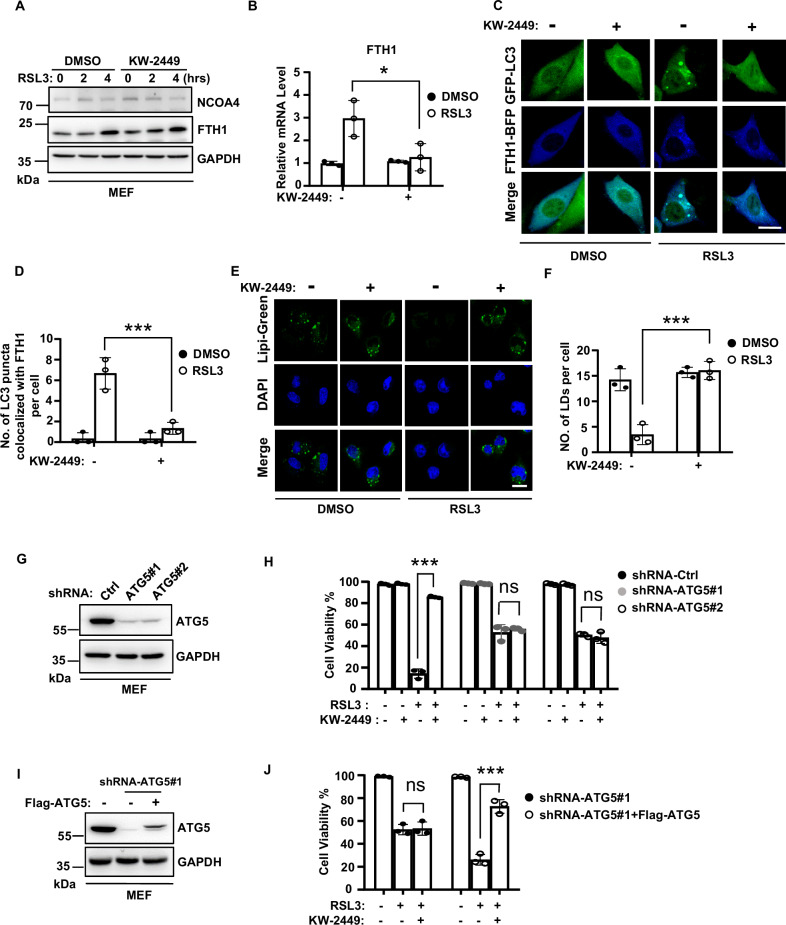
KW-2449 inhibits ferroptosis by targeting the autophagy pathway. **A** MEF cells were pre-treated with DMSO or KW-2449 for 30 min followed by treatment with RSL3 for the indicated time. Cells were lysed and immunoblotted with the NCOA4 and FTH1 antibodies. **B** MEF cells were pre-treated with DMSO or KW-2449 for 30 min followed by treatment with RSL3 for 2 h. Relative mRNA level of FTH1 was determined by real-time qPCR. **C** MEF cells stably expressed with GFP-LC3 and BFP-FTH1 were pre-treated with DMSO or KW-2449 for 30 min followed by treatment with RSL3 for 1 h. Representative confocal images of the cells were shown. Scale bar, 10 µm. **D** Statistic analysis of colocalization between GFP-LC3 and BFP-FTH1 in MEF cells from (**C**). **E** MEF cells were pre-treated with DMSO or KW-2449 for 30 min followed by treatment with RSL3 for 1 h. The cells were stained with Lipi-green probe to detect LDs. Representative confocal images of the cells were shown. Scale bar, 20 µm. **F** Statistic analysis of LDs formation in the MEF cells from (**E**). **G** ATG5 was knocked down by two shRNA lentiviruses in MEF cells. The expression of ATG5 was examined by Western blotting with ATG5 antibody. **H** shRNA-Control, shRNA-ATG5#1, and -ATG5#2 MEF cells were pre-treated with KW-2449 for 30 min followed by treatment with RSL3 for 6 h. Cell viability was determined by CellTiter-Lumi assay. **I** shRNA-ATG5#1 MEF cells were infected with lentivirus containing pLVX with Flag-tagged mouse ATG5 to reconstitute the expression of ATG5. The expression of ATG5 was examined by Western blotting with ATG5 antibody. **J** shRNA-ATG5#1 and shRNA-ATG5#1 with Flag-ATG5 expressed MEF cells were treated as in (**H**). Cell viability was determined by CellTiter-Lumi assay. Bar graphs represent the mean ± SD from three independent experiments. All Western data are representative of three independent experiments. Statistical analysis was performed using a two-sided student’s t-test. The levels of significance were indicated by *P < 0.05; ***P < 0.001; ns not significant.

In ferritinophagy, nuclear receptor coactivator 4 (NCOA4) acts as a cargo receptor to deliver ferritin for lysosomal degradation, which leads to the release of free iron and thus an increase of cellular labile iron pool (LIP) [39, 40]. We then examined the expression levels of NCOA4 and ferritin heavy chain 1 (FTH1), a substrate of ferritinophagy, in RSL3-treated cells. We found NCOA4 level did not change in RSL3-induced ferroptosis (Fig. 5A). However, FTH1 level was increased during ferroptosis and KW-2449 had no effect on the increase of FTH1(Fig. 5A). As previous studies demonstrated that the expression of FTH1 could be transcriptionally induced by LIP accumulation in ferroptosis [11, 41], we then measured the mRNA level of FTH1 by real-time (RT)-PCR. Consistent with a previous study, FTH1 mRNA was increased during RSL3-induced ferroptosis [11], while this increase was suppressed by KW-2449 (Fig. 5B). As we found KW-2449 decreased the amount of ferrous iron in ferroptosis, these data suggest that KW-2449 may inhibit the transcription of FTH1 by preventing LIP accumulation in ferroptosis. To test whether there was a simultaneous autophagic degradation of FTH1 protein upon ferroptosis induction, we examined the colocalization of GFP-LC3 and BFP (Blue fluorescence protein)-tagged-FTH1 in RSL3-induced ferroptosis. As shown in Fig. 5C, upon RSL3 treatment, BFP-FTH1 was co-localized with the cluster of GFP-LC3 puncta, indicating ferritinophagy was induced. In contrast, the colocalization between BFP-FTH1 and GFP-LC3 puncta was inhibited by KW-2449 treatment, suggesting KW-2449 inhibits autophagic turnover of FTH1 (Fig. 5C, D and Fig. S4). Additionally, we found all known targets of KW-2449, including FLT3, ABL, and Aurora kinases, were not involved in RSL3-induced ferritinophagy because the colocalization between BFP-FTH1 and GFP-LC3 puncta by RSL3 treatment was not affected by siRNA-mediated gene knockdown (Fig. S5). Thus, these data suggest that KW-2449 must target other unidentified factor(s) to inhibit RSL3-induced ferritinophagy.

In addition to ferritinophagy, other selective types of autophagy including lipophagy, clockophagy, or CMA were shown to promote ferroptosis [10]. Lipophagy is a selective type of autophagy that targets lipid droplets (LDs) for degradation [42]. In lipophagy, LDs are broken down by autolysosomes, which leads to the release of polyunsaturated fatty acids (PUFAs) to the membrane for oxidation [43]. It has been reported that lipophagy is induced in ferroptosis and the level of LDs is negatively related to the degree of ferroptosis [13]. We found the level of LDs, as examined by Lipi-Green dye, was significantly decreased in RSL3-induced ferroptosis, while KW-2449 restored the level of LDs in RSL3-treated cells (Fig. 5E, F), suggesting KW-2449 inhibit lipophagy in RSL3-induced ferroptosis. Clockophagy was reported to degrade a circadian transcription factor ARNTL in RSL3-induced ferroptosis. ARNTL degradation promotes ferroptosis by upregulating the transcription of EGLN2 to activate the pro-survival transcription factor HIF1A [14]. We did not observe the degradation of ARNTL in RSL3-treated MEFs, however, KW-2449 suppressed RSL3-induced upregulation of EGLN2 mRNA in MEFs (Fig. S6A, B). Additionally, it has been reported that CMA was involved in the execution of ferroptosis by promoting the degradation of GPX4 [15]. We found that KW-2449 did not prevent the degradation of GPX4 in RSL3-induced ferroptosis (Fig. S6C), suggesting KW-2449 has no effect on CMA-mediated degradation of GPX4 in ferroptosis.

Our data indicate that KW-2449 acts as an autophagy inhibitor to block selective autophagy, including ferritinophagy and lipophagy that are involved in ferroptosis execution. To further confirm that autophagy is the target of KW-2449 to inhibit ferroptosis, we then knocked down Atg5, an essential gene for autophagosome formation [44], to block autophagy in MEFs (Fig. 5G). As autophagy promotes ferroptosis [11, 12], RSL3-induced cell death was delayed by knockdown of Atg5 (Fig. S7A). At a later time, this type of cell death could be suppressed by ferroptosis inhibitor Fer-1, suggesting RSL3-induced cell death in Atg5 knockdown cells is ferroptosis (Fig. S7B). Remarkably, compared to shRNA-control MEFs, KW-2449 no more protected Atg5 knockdown cells from RSL3-induced ferroptosis (Fig. 5H). Furthermore, when Atg5 expression was reconstituted in these cells, the protective ability of KW-2449 on RSL3-induced ferroptosis was re-exhibited (Fig. 5I, J). Thus, these data suggest that KW-2449 targets the autophagy pathway to inhibit ferroptosis.

### KW-2449 targets ULK1 to inhibit autophagy

**Fig. 6:**
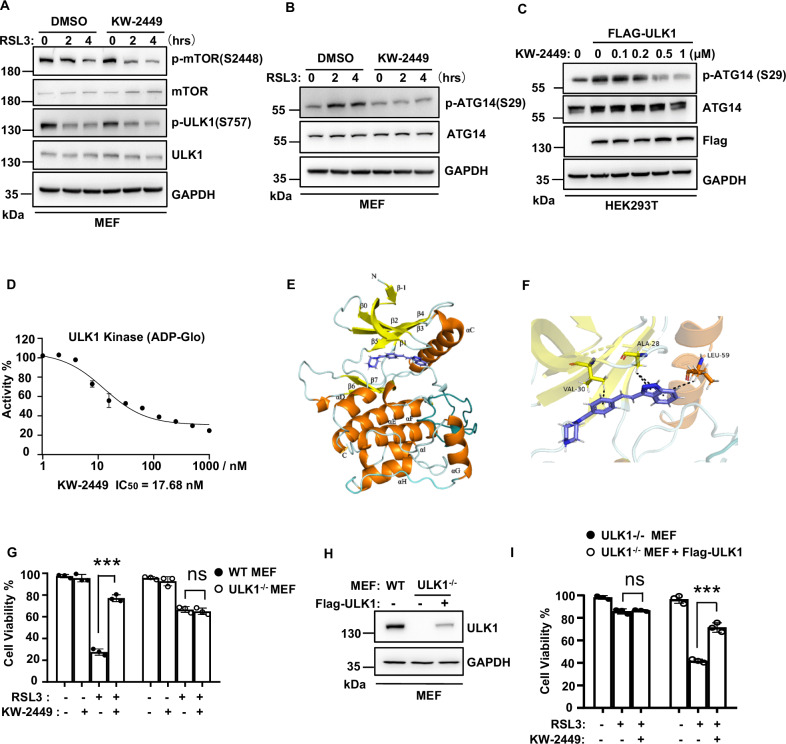
KW-2449 targets ULK1 to inhibit autophagy. **A**, **B** MEF cells were pre-treated with DMSO or KW-2449 for 30 min followed by treatment with RSL3 for the indicated time. Cells were lysed and immunoblotted with indicated antibodies. **C** HEK293T cells were transfected with empty vector or Flag-tagged human ULK1. After 16 h, cells were treated with KW-2449 at indicated concentrations for 8 h. Cells were then lysed and immunoblotted with indicated antibodies. **D** Quantification of ADP-Glo kinase assays performed with recombinant human ULK1 in the presence of increasing concentrations of KW-2449. **E** Overall binding model of ULK1 with KW-2449. KW-2449 is shown in blue. α**-**helix is shown in orange. β-sheet is shown in yellow. **F** Closeup view of KW-2449 bound to ULK1. The π−alkyl interactions were indicated by the black line. Key residues that contact KW-2449 are labeled. **G** Paired WT and ULK1-deficient MEF cells were pre-treated with DMSO or KW-2449 for 30 min. Then, WT MEF cells were treated with RSL3 for 6 h and ULK1-deficient MEF cells were treated with RSL3 for 24 h, respectively. Cell viability was analyzed by CellTiter-Lumi assay. **H** ULK1-deficient MEF cells were infected with the lentivirus containing the pLVX with Flag-tagged mouse ULK1 to reconstitute the expression of ULK1. The expression of ULK1 was examined by Western blotting with ULK1 antibody. **I** ULK1-deficient and ULK1-deficient with Flag-ULK1 expressed MEF cells were pre-treated with DMSO or KW-2449 for 30 min followed by treatment with RSL3 for 6 h. Cell viability was analyzed by CellTiter-Lumi assay. Bar graphs represent the mean ± SD from three independent experiments. All Western data are representative of three independent experiments. Statistical analysis was performed using a two-sided student’s t-test. The levels of significance were indicated by ***P < 0.001; ns not significant.

We next want to elucidate the inhibitory mechanism of KW-2449 in autophagy. In nutrient deprivation-induced autophagy, mTOR (mechanistic target of rapamycin kinase) is a key upstream regulator to modulate ULK1 kinase activity [45, 46]. We found that mTOR phosphorylation at Ser2448 and ULK1 phosphorylation at Ser757 were both decreased by RSL3 treatment (Fig. 6A), indicating autophagy signaling is activated [47]. However, KW-2449 did not affect phosphorylation levels of mTOR and ULK1 at these sites (Fig. 6A). We then determined the levels of ULK1 downstream signaling regulators in autophagy induction. The active ULK1 directly phosphorylates ATG14 at Ser 29 to promote autophagy induction and maturation [48]. We found KW-2449 inhibited RSL3-induced phosphorylation of ATG14 at Ser 29 in MEFs (Fig. 6B). Similar results were also obtained in AA starvation-induced autophagy in MEFs (Fig. S8A) and RSL3-induced autophagy in human HT29 cells (Fig. S8B). As ULK1 is the direct kinase for ATG14 [48] and KW-2449 was previously identified as a multi-targeted kinase inhibitor [35], we then tested whether KW-2449 was able to suppress ULK1 kinase activity. We found overexpression of ULK1 increased the phosphorylation of endogenous ATG14 in HEK293 cells, while KW-2449 dose-dependently inhibited the phosphorylation of ATG14 (Fig. 6C). We then performed an in vitro kinase assay with recombinant ULK1 protein and found KW-2449 strongly inhibited the kinase activity of ULK1 with IC_50_ (half maximal inhibitory concentrations) 17.68 nM (Fig. 6D). Thus, these data indicate that KW-2449 is a potent ULK1 kinase inhibitor. To evaluate the binding pattern of ULK1 with KW-2499, we generated a docked model of ULK1 with KW-2449 based on the co-crystal structure of ULK1 with its inhibitor (PDB ID: 4WNO) [49]. As shown in Fig. 6E, KW-2449 was docked into the ATP-binding pocket of ULK1 and could form π−alkyl interactions with residues ALA28, VAL30, and LEU59 of ULK1 (Fig. 6F).

To confirm whether ULK1 is the direct target of KW-2449 in ferroptosis, we employed ULK1 knockout MEFs to examine the inhibitory activity of KW-2449 in ferroptosis. We found RSL3-induced LC3-II accumulation (Fig. S9), ferritinophagy (Fig. S10A, B), and lipophagy (Fig. S11A, B) were all inhibited by ULK1 knockout, suggesting ULK1 is required for RSL3-induced autophagy. As ULK1 depletion blocks autophagy initiation, we found that knockout of ULK1 significantly delayed RSL3-induced ferroptosis compared to WT cells (Fig. S12). Remarkably, KW-2449 no more protected ULK1 knockout cells from RSL3-induced ferroptosis (Fig. 6G). Furthermore, when ULK1 expression was reconstituted in these cells, the inhibitory activity of KW-2449 on RSL3-induced cell death (Fig. 6H, I), ferrotinphagy (Fig. S10C, D) and lipophagy (Fig. S11C, D) was re-established. Taken together, these data suggest that KW-2449 functions as a ULK1 kinase inhibitor to block autophagy in ferroptosis.

### KW-2449 protects mice against iron overload-induced multiple organ dysfunction syndrome (MODS)

**Fig. 7:**
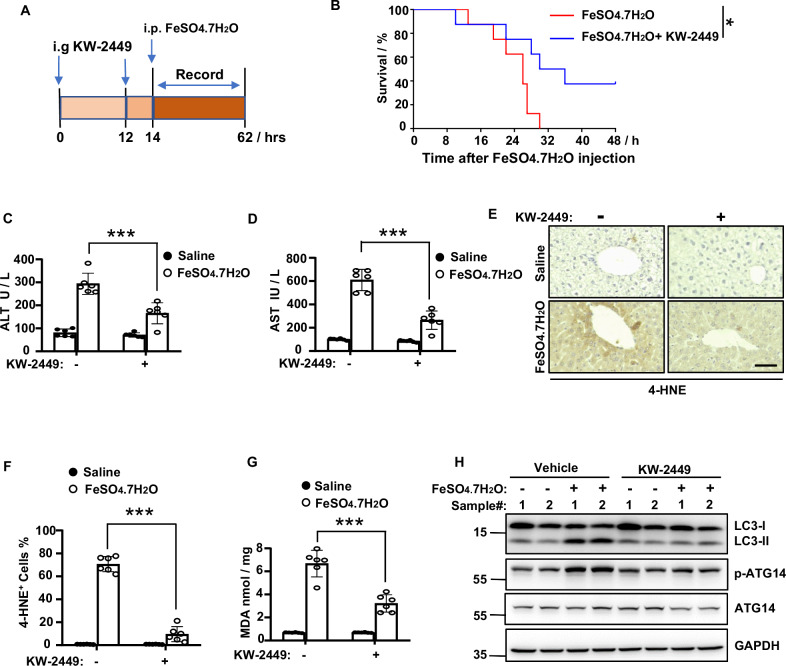
KW-2449 protects mice against iron overload-induced MODS. **A** Schematic representation of the KW-2449 treatment regime in the experimental model of MODS. **B** Survival curve of the mice treated with vehicle control or KW-2449 in the experimental model of MODS. **C** Mice were treated as in (**A**). After 4 h, the serum levels of ALT and **D** AST were determined. **E** Representative images of immunohistochemical staining of 4-HNE in liver tissues from the mice in (**C**). Scale bar, 50 µm. **F** The percentage of 4-HNE-positive cells in (**E**). **G** The levels of MDA in liver homogenate from the mice in (**C**). **H** Western blot analysis of LC3, p-ATG14, and ATG14 in freshly isolated livers from the mice in (**C**). Bar graphs represent the mean ± SD from three independent experiments. All Western data are representative of three independent experiments. Statistical analysis was performed using a two-sided student’s t-test. The levels of significance were indicated by *P < 0.05; ***P < 0.001.

Previous study demonstrates that the severity of multiorgan dysfunction in clinic patients is related to plasma catalytic iron and lipid peroxidation [28]. The experimental mice models of iron overload-induced MODS could mimic the complexity of clinical MODS. Importantly, ferroptosis, rather than necroptosis, parthanatos, or cyclophilin D-dependent necrosis, is primarily responsible for organ damage in this model [28]. Therefore, to gain more insights into the protective role of KW-2449 in ferroptosis-related diseases, we established a mouse model of iron overload-induced MODS (Fig. 7A). We found that oral gavage (i.g.) of KW-2449 protected mice from iron overload-induced death (Fig. 7B). In the case of ferroptosis-driven acute liver injury, KW-2449 significantly ameliorated the increase of AST and ALT in mice models of MODS (Fig. 7C, D). Furthermore, the increase of two lipid peroxidation markers induced by iron overload, 4-HNE and MDA, were both inhibited by KW-2449 (Fig. 7E–G), indicating KW-2449 prevents ferroptosis in mice models of MODS.

We then investigated the effects of KW-2449 on autophagy in MODS. By examining the expression of LC3-II and phosphorylation of ATG14, we found iron overload significantly induced the accumulation of LC3-II and phosphorylation of ATG14 in the liver (Fig. 7H). In contrast, KW-2449 prevented the accumulation of LC3-II and phosphorylation of ATG14 induced by iron overload (Fig. 7H). Taken together, these data indicate that KW-2449 protects mice against iron overload-induced MODS by inhibiting ferroptosis-associated autophagy.

### Nec-1 targets the autophagy pathway to inhibit ferroptosis

**Fig. 8:**
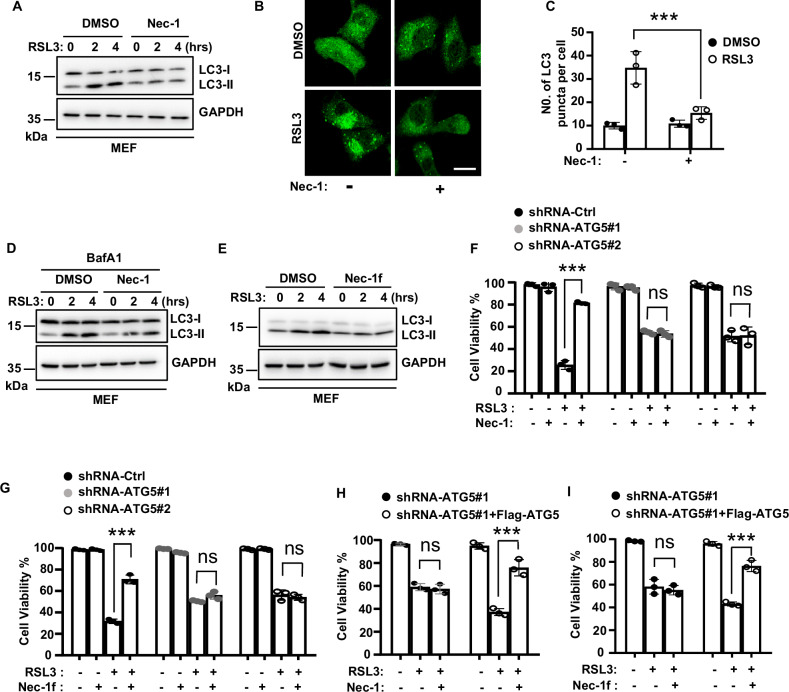
Nec-1 targets the autophagy pathway to inhibit ferroptosis. **A** MEF cells were pre-treated with DMSO or Nec-1 for 30 min followed by treatment with RSL3 for the indicated time. Cells were lysed and immunoblotted with LC3 antibody. **B** MEF cells stably expressed GFP-LC3 were pre-treated with DMSO or Nec-1 for 30 min followed by treatment with RSL3 for 1 h. Representative confocal images of the cells were shown. Scale bar, 20 µm. **C** Statistic analysis of LC3 puncta formation in MEF cells from (**B**). **D** MEF cells were pre-treated with DMSO or Nec-1 plus BafA1 for 30 min followed by treatment with RLS3 for the indicated time. Cells were lysed and immunoblotted with LC3 antibody. **E** MEF cells were pre-treated with DMSO or Nec-1f for 30 min followed by treatment with RSL3 for the indicated time. Cells were lysed and immunoblotted with LC3 antibody. **F** shRNA-Control, shRNA-ATG5#1, and -ATG5#2 MEF cells were pre-treated with Nec-1 or **G** Nec-1f for 30 min followed by treatment with RSL3 for 6 h. Cell viability was determined by CellTiter-Lumi assay. **H** shRNA-ATG5#1 and shRNA-ATG5#1 with Flag-ATG5 expressed MEF cells were pre-treated with Nec-1 or **I** Nec-1f for 30 min followed by treatment with RSL3 for 6 h. Cell viability was determined by CellTiter-Lumi assay. Bar graphs represent the mean ± SD from three independent experiments. All Western data are representative of three independent experiments. Statistical analysis was performed using a two-sided student’s t-test. The levels of significance were indicated by ***P < 0.001; ns not significant.

Previous studies demonstrated that Nec-1, a RIPK1-targeted inhibitor of necroptosis, and its derivative Nec-1f also inhibited ferroptosis at relatively higher concentrations [22–24]. Consistent with these studies, we observed that Nec-1 inhibited RSL3-induced ferroptosis in MEFs, although the IC_50_ of Nec-1 for ferroptosis inhibition was approximately 7-fold greater than its IC_50_ for necroptosis inhibition (Fig. S13). Since Nec-1 is able to inhibit autophagy in TNF-induced necroptosis [50], we then asked whether Nec-1 inhibited ferroptosis by targeting the autophagy pathway. We found Nec-1 inhibited RSL3-induced increase of LC3-II and the formation of GFP-LC3 puncta in MEFs (Fig. 8A–C). Furthermore, when cells were treated with BafA1 to block lysosomal degradation of autophagosomes, Nec-1 still inhibited the increase of LC3-II induced by RSL3 treatment (Fig. 8D), suggesting Nec-1 prevents RSL3-induced autophagosome formation. Similarly, we found Nec-1f also inhibited RSL3-induced increase of LC3-II in MEFs (Fig. 8E).

To confirm whether autophagy is the target of Nec-1 to inhibit ferroptosis, we used Atg5 knockdown MEFs to block autophagy and found that both Nec-1 and Nec-1f did not protect cells from ferroptosis in Atg5 knockdown cells (Fig. 8F, G). However, the protective abilities of Nec-1 and Nec-1f on ferroptosis were re-exhibited when Atg5 expression was reconstituted in these cells (Fig. 8H, I). Thus, these data suggest that Nec-1 and its derivative Nec-1f target the autophagy pathway to inhibit ferroptosis. Additionally, unlike KW-2449, Nec-1 is not a kinase inhibitor of ULK1 (Fig. S14), suggesting Nec-1 may target other molecular(s) in the autophagy pathway to inhibit ferroptosis.

## Discussion

As different forms of RCD can occur together to mediate pathology, targeting complex diseases with multiple dysregulated cell death pathways by a single compound may have several advantages in drug discovery, including enhancing synergistic effects, simplifying the treatment regimens, minimizing the potential for drug interactions and reducing the costs of drug development [4, 51]. Here we show that KW-2499, a previously described necroptosis inhibitor, also inhibits ferroptosis with an EC_50_ in the nM range. KW-2449 is originally identified as a multi-targeted kinase inhibitor to induce cytotoxicity in leukemia cells [35]. We previously demonstrated that KW-2449 is a necroptosis inhibitor by targeting RIPK1 kinase activity [27]. Our present data reveal that KW-2449 inhibited ferroptosis by blocking ULK1 kinase activity in autophagy. Therefore, these studies indicate that KW-2449 suppresses both necroptosis and ferroptosis by blocking RIPK1 activity in the necroptosis signaling pathway and ULK1 activity in the autophagy signaling pathway (Fig. S15). Unlike classic ferroptosis inhibitors Fer-1 and liprostatin-1, which are free-radical trapping antioxidants, KW-2449 has high metabolic stability after oral administration [52]. We found that oral gavage of KW-2449 protected mice against cisplatin-induced AKI and iron overload-induced MODS, suggesting its potential for therapeutic application in complex necrosis-related diseases. In our experiments, KW-2449 exhibited low cytotoxicity over the range of concentrations and treatment times showing blockade of ferroptosis and necroptosis [27]. However, KW-2449 is able to induce cytotoxicity in cancer cells by targeting FLT3, ABL, and Aurora kinases [35, 53]. The lack of kinase specificity of KW-2449 could increase its toxicity and limit its efficacy against ferroptosis and necroptosis. Therefore, in a future study, it is necessary to determine the co-crystal structures of KW-2449 with ULK1 and RIPK1, and perform structure–activity relationship studies in order to develop novel dual inhibitors of ferroptosis and necroptosis with improved specificity and efficacy.

Although autophagy is often observed in cells undergoing RCD, the relationship between autophagy and cell death is complex and can vary depending on the specific cellular context and the type of cell death involved [54]. Recently, autophagy is recognized as a part of ferroptosis execution mechanism [10, 11]. It has been shown that autophagic flux is increased in ferroptosis and autophagy-deficient cells are resistant to ferroptotic insults [11, 12]. Mechanistically, autophagy promotes the selective degradation of anti-ferroptotic proteins including GPX4 [15, 55], ferritin [11, 12], ARNTL [14], SLC40A1 [56], CDH2 [57] or LDs [13], thereby increasing iron accumulation and lipid peroxidation. Although the close crosstalk and interdependence between autophagy and ferroptosis has been well documented, it is worth noticing that not all the autophagy inhibitors prevent ferroptosis, which is most likely due to the off-targets of autophagy inhibitors [12]. The role of autophagy in necroptosis is complex and context-dependent. Autophagy may have no effect or either pro- or anti-death effects on necroptosis, depending on the cellular context and the specific conditions [50, 58–61]. Our data suggest that KW-2449 is a ULK1 kinase inhibitor to target autophagy in ferroptosis and AA-deprived conditions, which is the action mechanism of KW-2449 to inhibit ferroptosis.

There are four necroptosis inhibitors that have been reported to inhibit ferroptosis, including a RIPK1-targeted necroptosis inhibitor Nec-1 [22] and its derivative Nec-1f [28], a plant derivative nigratine [25] (also known as 6E11) and an HSP90 inhibitor CDDO [15] (also known as bardoxolone). Among these inhibitors, Nec-1 was previously reported to inhibit autophagy during TNF-induced necroptosis [50]. Our study indicates that Nec-1 and its derivative Nec-1f also inhibit autophagy in ferroptosis, which is a prerequisite for these inhibitors to prevent ferroptosis (Extended Data Fig. 9). Unlike KW-2449, we found that Nec-1 is not a kinase inhibitor of ULK1. At the present stage, the detailed mechanism of Nec-1 to inhibit ferroptosis-associated autophagy is unknown, and future studies are needed to identify the potential target (s) of Nec-1 in the autophagy pathway. Collectively, our data suggest that the autophagy pathway can be a common target for necroptosis inhibitors to prevent ferroptosis. Therefore, further modification of necroptosis inhibitors to improve their efficacy on autophagy inhibition could be a promising strategy to develop dual inhibitors of necroptosis and ferroptosis in clinical trials.

In summary, our identification of KW-2449 as a dual inhibitor of necroptosis and ferroptosis provides the first understanding of how necroptosis inhibitors can prevent ferroptosis. This study also reveals a potential mechanism by which the classic necroptosis inhibitor Nec-1 is able to suppress ferroptosis. Our work suggests that autophagy is a targetable pathway for necroptosis inhibitors to prevent ferroptosis, which provides a theoretical basis for developing dual inhibitors of necroptosis and ferroptosis in future clinical applications.

## Supplementary information

Supplementary Fig. 1 - Supplementary Fig. 15

Supplementary table 1

Supplementary table 2

Raw data of WB

## Data Availability

The authors declare that all data supporting the findings of this study are available within the article and the Supplementary Information. All other data are available from the corresponding authors upon request.
