# Correction: Identification of KW-2449 as a dual inhibitor of ferroptosis and necroptosis reveals that autophagy is a targetable pathway for necroptosis inhibitors to prevent ferroptosis

**DOI:** 10.1038/s41419-025-07705-x

**Published:** 2025-05-12

**Authors:** Yaxing Zhao, Qingsong Wang, Jing Zhu, Jin Cai, Xiaona Feng, Qianqian Song, Hui Jiang, Wenqing Ren, Yuan He, Ping Wang, Du Feng, Jianqiang Yu, Yue Liu, Qihui Wu, Siriporn Jitkaew, Zhenyu Cai

**Affiliations:** 1 Tongji University Cancer Center, Shanghai Tenth People’s Hospital, School of Medicine, Tongji University, Shanghai, China; 2 College of Pharmacy, Ningxia Medical University, Yinchuan, Ningxia Hui Autonomous Region, Yinchuan, China; 3 Department of Biochemistry and Molecular Biology, School of Medicine, Tongji University, Shanghai, China; 4 Guangzhou Municipal and Guangdong Provincial Key Laboratory of Protein Modification and Degradation, School of Basic Medical Sciences, Guangzhou Medical University, Guangzhou, China; 5 Shanghai Fourth People’s Hospital, School of Medicine, Tongji University, Shanghai, China; 6 Center of Excellence for Cancer and Inflammation, Department of Clinical Chemistry, Faculty of Allied Health Sciences, Chulalongkorn University, Bangkok, Thailand; 7 State Key Laboratory of Cardiology and Medical Innovation Center, Shanghai East Hospital, School of Medicine, Tongji University, Shanghai, China

**Keywords:** Necroptosis, Macroautophagy, Kinases

Correction to: *Cell Death and Disease* 10.1038/s41419-024-07157-9, published online 21 October 2024

In this article the author name and family name have been interchanged: “Jitkaew Siriporn” should be read “Siriporn Jitkaew”.

The original article has been corrected.

