# Supplementary material for: Identification of KW-2449 as a dual inhibitor of ferroptosis and necroptosis reveals that autophagy is a targetable pathway for necroptosis inhibitors to prevent ferroptosis
Source: Cell Death Dis. 2024 Oct 21;15(10):764. doi: 10.1038/s41419-024-07157-9 (PMC11493980; doi:10.1038/s41419-024-07157-9)
Supplement: Supplementary file 4 — Raw data of WB [file 41419_2024_7157_MOESM4_ESM.pptx]

## Slide 1
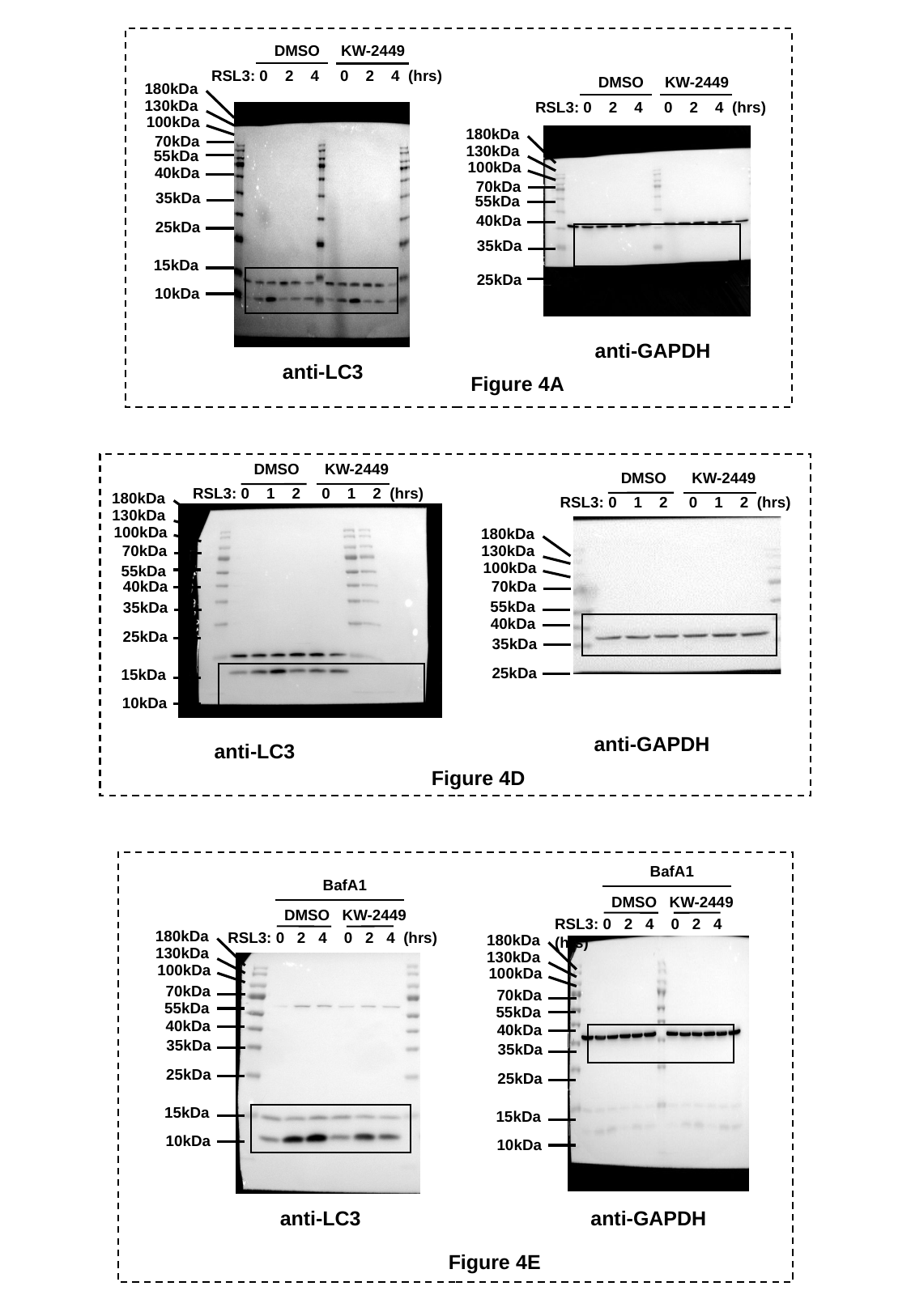

DMSO KW-2449
RSL3: 0 2 4 0 2 4 (hrs)
DMSO KW-2449
180kDa
130kDa
RSL3: 0 2 4 0 2 4 (hrs)
100kDa
180kDa
70kDa
130kDa
55kDa
100kDa
40kDa
70kDa
35kDa
55kDa
40kDa
25kDa
35kDa
15kDa
25kDa
10kDa
anti-GAPDH
anti-LC3
Figure 4A
DMSO KW-2449
DMSO KW-2449
RSL3: 0 1 2 0 1 2 (hrs)
180kDa
RSL3: 0 1 2 0 1 2 (hrs)
130kDa
100kDa
180kDa
70kDa
130kDa
100kDa
55kDa
40kDa
70kDa
55kDa
35kDa
40kDa
25kDa
35kDa
25kDa
15kDa
10kDa
anti-GAPDH
anti-LC3
Figure 4D
BafA1
BafA1
DMSO KW-2449
DMSO KW-2449
RSL3: 0 2 4 0 2 4 (hrs)
180kDa
RSL3: 0 2 4 0 2 4 (hrs)
180kDa
130kDa
130kDa
100kDa
100kDa
70kDa
70kDa
55kDa
55kDa
40kDa
40kDa
35kDa
35kDa
25kDa
25kDa
15kDa
15kDa
10kDa
10kDa
anti-LC3
anti-GAPDH
Figure 4E

## Slide 2
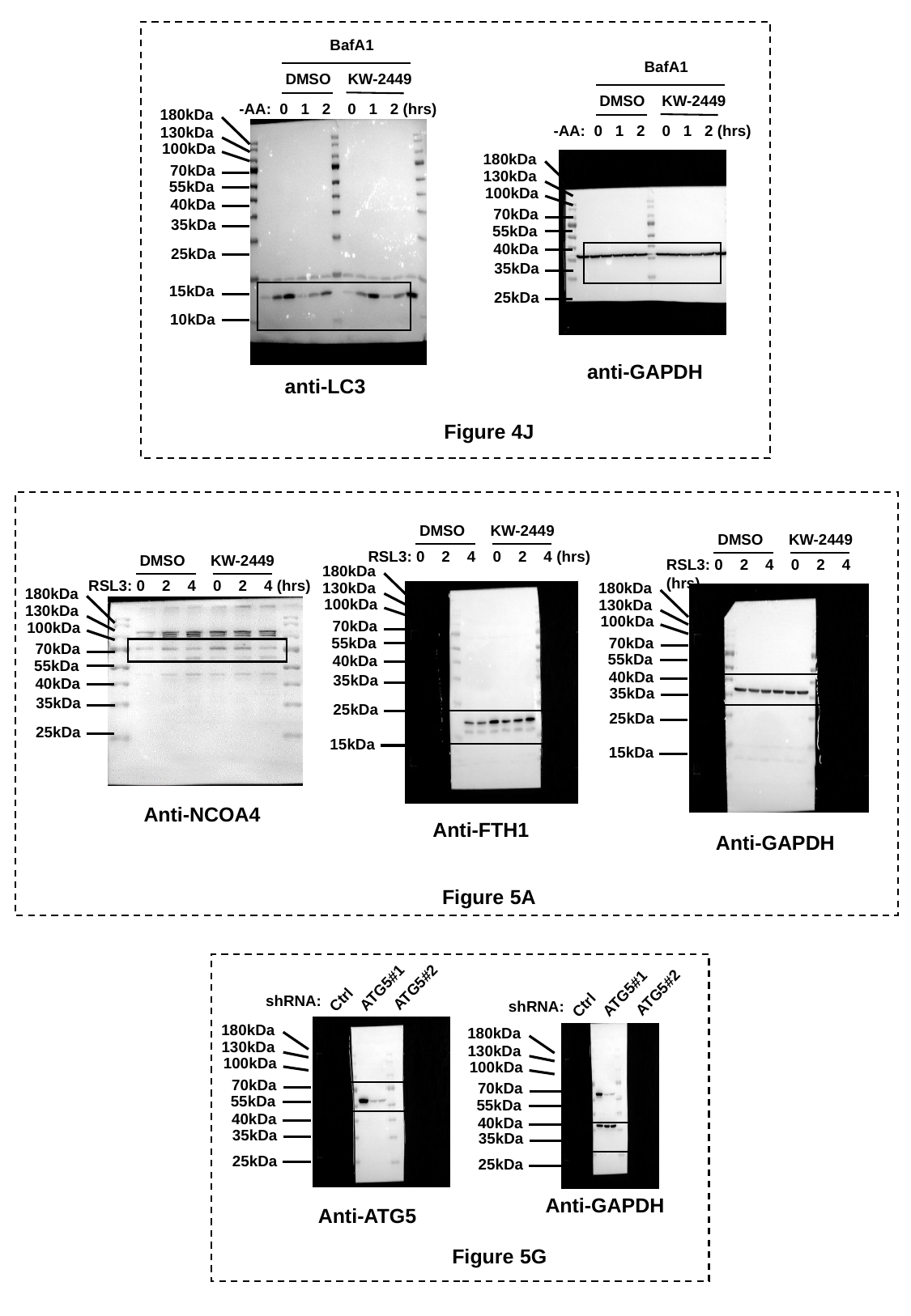

BafA1
BafA1
DMSO KW-2449
DMSO KW-2449
-AA: 0 1 2 0 1 2 (hrs)
180kDa
-AA: 0 1 2 0 1 2 (hrs)
130kDa
100kDa
180kDa
70kDa
130kDa
55kDa
100kDa
40kDa
70kDa
35kDa
55kDa
40kDa
25kDa
35kDa
15kDa
25kDa
10kDa
anti-GAPDH
anti-LC3
Figure 4J
DMSO KW-2449
DMSO KW-2449
RSL3: 0 2 4 0 2 4 (hrs)
DMSO KW-2449
RSL3: 0 2 4 0 2 4 (hrs)
180kDa
RSL3: 0 2 4 0 2 4 (hrs)
180kDa
130kDa
180kDa
100kDa
130kDa
130kDa
100kDa
70kDa
100kDa
55kDa
70kDa
70kDa
55kDa
40kDa
55kDa
40kDa
35kDa
40kDa
35kDa
35kDa
25kDa
25kDa
25kDa
15kDa
15kDa
Anti-NCOA4
Anti-FTH1
Anti-GAPDH
Figure 5A
ATG5#1
ATG5#2
ATG5#1
ATG5#2
Ctrl
shRNA:
Ctrl
shRNA:
180kDa
180kDa
130kDa
130kDa
100kDa
100kDa
70kDa
70kDa
55kDa
55kDa
40kDa
40kDa
35kDa
35kDa
25kDa
25kDa
Anti-GAPDH
Anti-ATG5
Figure 5G

## Slide 3
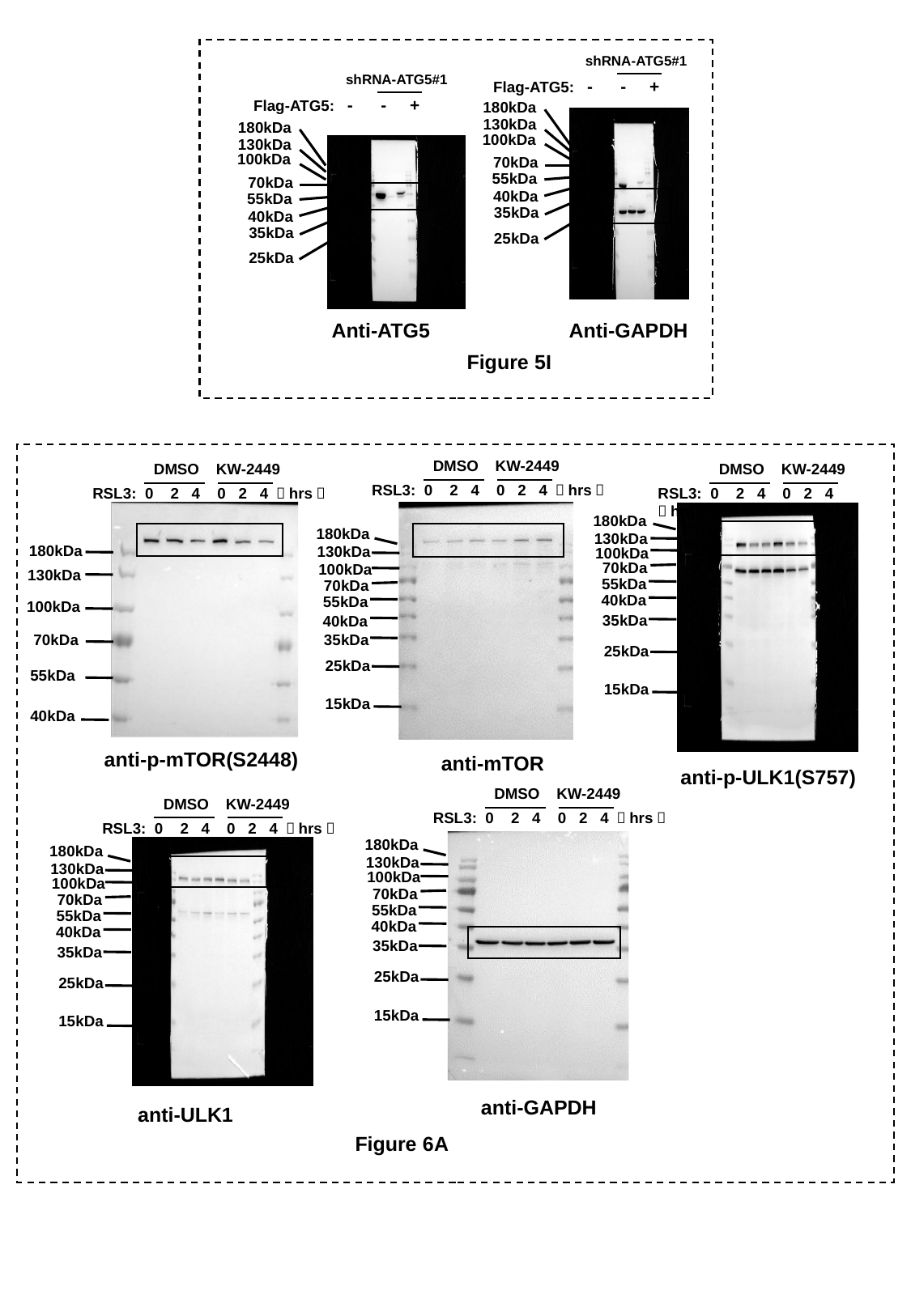

shRNA-ATG5#1
shRNA-ATG5#1
Flag-ATG5: - - +
Flag-ATG5: - - +
180kDa
130kDa
180kDa
100kDa
130kDa
100kDa
70kDa
55kDa
70kDa
40kDa
55kDa
35kDa
40kDa
35kDa
25kDa
25kDa
Anti-GAPDH
Anti-ATG5
Figure 5I
DMSO KW-2449
DMSO KW-2449
DMSO KW-2449
RSL3: 0 2 4 0 2 4 （hrs）
RSL3: 0 2 4 0 2 4 （hrs）
RSL3: 0 2 4 0 2 4 （hrs）
180kDa
180kDa
130kDa
180kDa
130kDa
100kDa
70kDa
100kDa
130kDa
55kDa
70kDa
40kDa
55kDa
100kDa
35kDa
40kDa
35kDa
70kDa
25kDa
25kDa
55kDa
15kDa
15kDa
40kDa
anti-p-mTOR(S2448)
anti-mTOR
anti-p-ULK1(S757)
DMSO KW-2449
DMSO KW-2449
RSL3: 0 2 4 0 2 4 （hrs）
RSL3: 0 2 4 0 2 4 （hrs）
180kDa
180kDa
130kDa
130kDa
100kDa
100kDa
70kDa
70kDa
55kDa
55kDa
40kDa
40kDa
35kDa
35kDa
25kDa
25kDa
15kDa
15kDa
anti-GAPDH
anti-ULK1
Figure 6A

## Slide 4
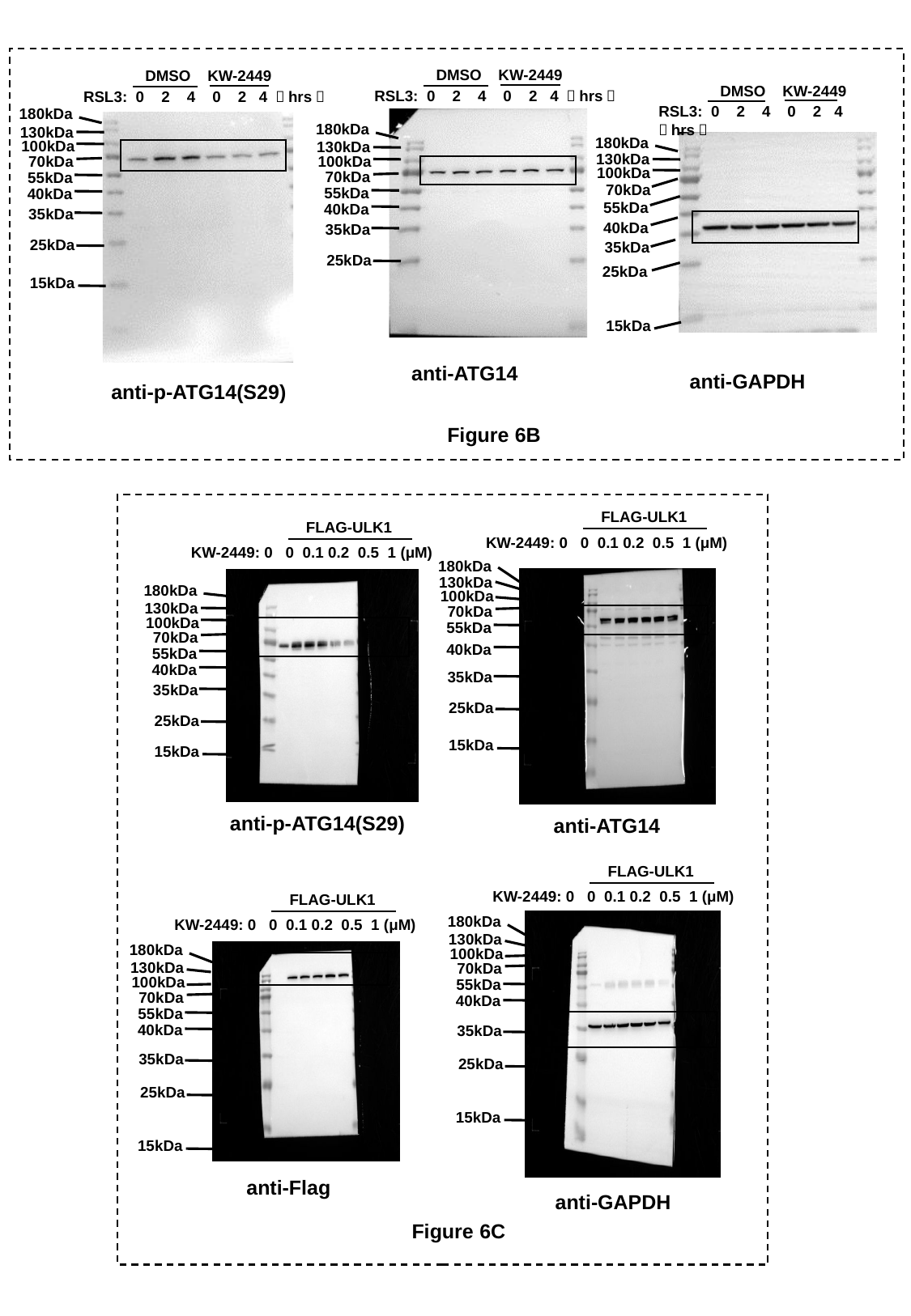

DMSO KW-2449
DMSO KW-2449
DMSO KW-2449
RSL3: 0 2 4 0 2 4 （hrs）
RSL3: 0 2 4 0 2 4 （hrs）
RSL3: 0 2 4 0 2 4 （hrs）
180kDa
180kDa
130kDa
180kDa
100kDa
130kDa
130kDa
70kDa
100kDa
100kDa
70kDa
55kDa
70kDa
55kDa
40kDa
55kDa
40kDa
35kDa
40kDa
35kDa
25kDa
35kDa
25kDa
25kDa
15kDa
15kDa
anti-ATG14
anti-GAPDH
anti-p-ATG14(S29)
Figure 6B
FLAG-ULK1
FLAG-ULK1
KW-2449: 0 0 0.1 0.2 0.5 1 (μM)
KW-2449: 0 0 0.1 0.2 0.5 1 (μM)
180kDa
130kDa
180kDa
100kDa
130kDa
70kDa
100kDa
55kDa
70kDa
40kDa
55kDa
40kDa
35kDa
35kDa
25kDa
25kDa
15kDa
15kDa
anti-p-ATG14(S29)
anti-ATG14
FLAG-ULK1
KW-2449: 0 0 0.1 0.2 0.5 1 (μM)
FLAG-ULK1
180kDa
KW-2449: 0 0 0.1 0.2 0.5 1 (μM)
130kDa
180kDa
100kDa
130kDa
70kDa
100kDa
55kDa
70kDa
40kDa
55kDa
40kDa
35kDa
35kDa
25kDa
25kDa
15kDa
15kDa
anti-Flag
anti-GAPDH
Figure 6C

## Slide 5
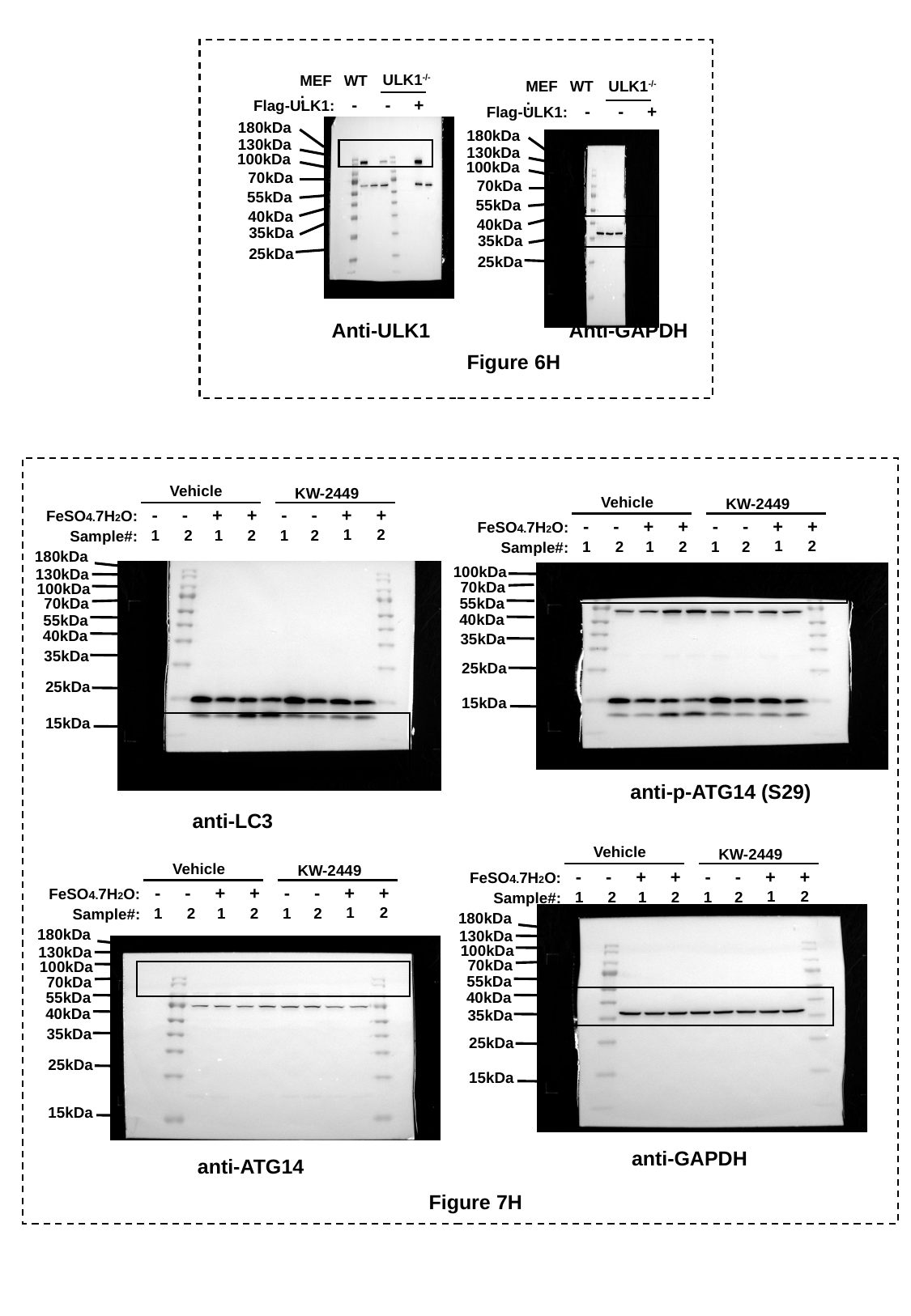

Flag-ULK1: - - +
Flag-ULK1: - - +
180kDa
180kDa
130kDa
130kDa
100kDa
100kDa
70kDa
70kDa
55kDa
55kDa
40kDa
40kDa
35kDa
35kDa
25kDa
25kDa
Anti-GAPDH
Anti-ULK1
Figure 6H
ULK1-/-
MEF:
WT
ULK1-/-
MEF:
WT
Vehicle
KW-2449
Vehicle
KW-2449
FeSO4.7H2O: - - + + - - + +
FeSO4.7H2O: - - + + - - + +
1
2
1
2
1
2
1
2
Sample#:
1
2
1
2
1
2
1
2
Sample#:
180kDa
100kDa
130kDa
70kDa
100kDa
55kDa
70kDa
40kDa
55kDa
40kDa
35kDa
35kDa
25kDa
25kDa
15kDa
15kDa
anti-p-ATG14 (S29)
anti-LC3
Vehicle
KW-2449
Vehicle
KW-2449
FeSO4.7H2O: - - + + - - + +
FeSO4.7H2O: - - + + - - + +
1
2
1
2
1
2
1
2
Sample#:
1
2
1
2
1
2
1
2
Sample#:
180kDa
180kDa
130kDa
100kDa
130kDa
70kDa
100kDa
55kDa
70kDa
40kDa
55kDa
40kDa
35kDa
35kDa
25kDa
25kDa
15kDa
15kDa
anti-GAPDH
anti-ATG14
Figure 7H

## Slide 6
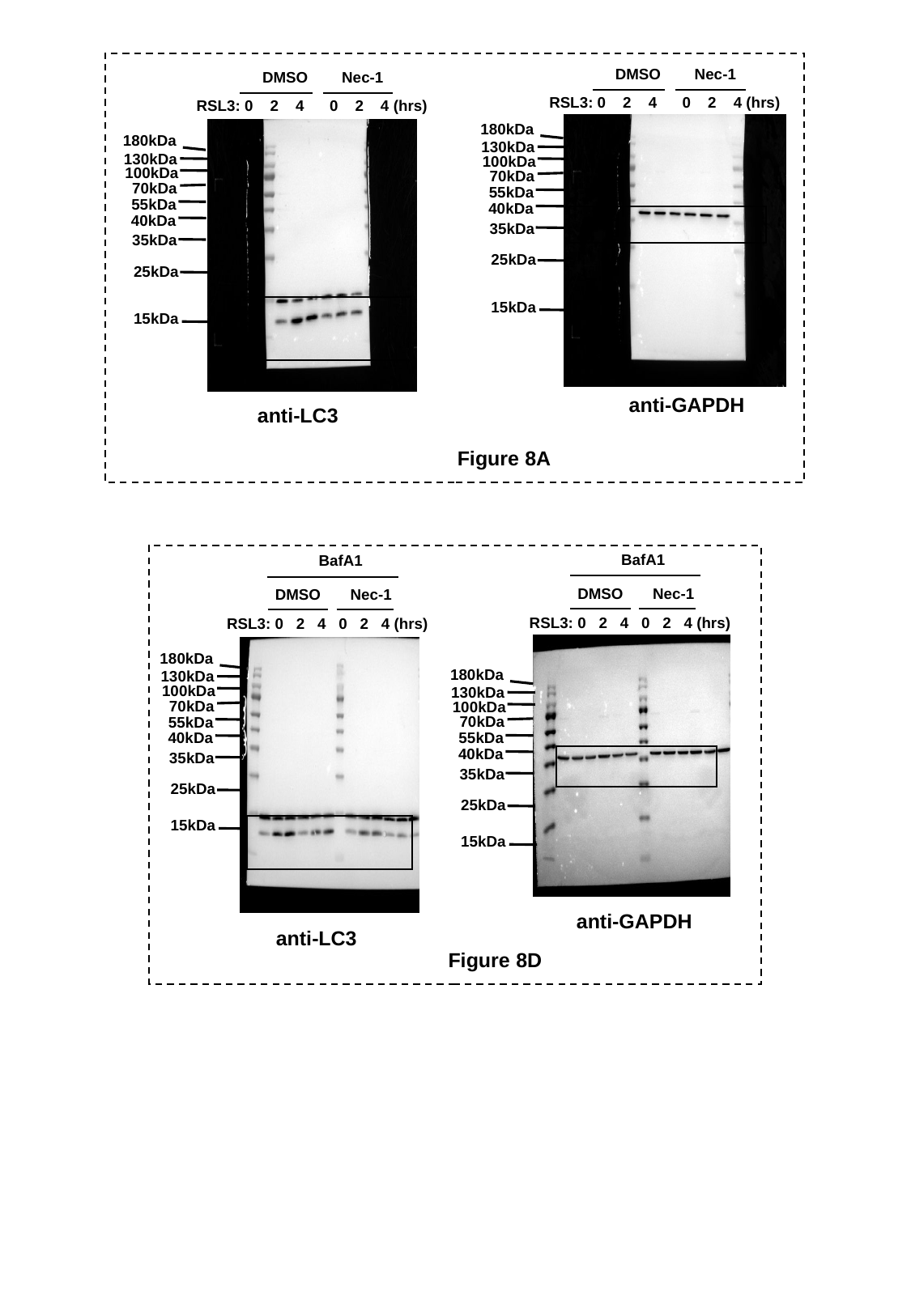

DMSO Nec-1
DMSO Nec-1
RSL3: 0 2 4 0 2 4 (hrs)
RSL3: 0 2 4 0 2 4 (hrs)
180kDa
180kDa
130kDa
130kDa
100kDa
100kDa
70kDa
70kDa
55kDa
55kDa
40kDa
40kDa
35kDa
35kDa
25kDa
25kDa
15kDa
15kDa
anti-GAPDH
anti-LC3
Figure 8A
BafA1
BafA1
DMSO Nec-1
DMSO Nec-1
RSL3: 0 2 4 0 2 4 (hrs)
RSL3: 0 2 4 0 2 4 (hrs)
180kDa
180kDa
130kDa
100kDa
130kDa
70kDa
100kDa
70kDa
55kDa
40kDa
55kDa
40kDa
35kDa
35kDa
25kDa
25kDa
15kDa
15kDa
anti-GAPDH
anti-LC3
Figure 8D

## Slide 7
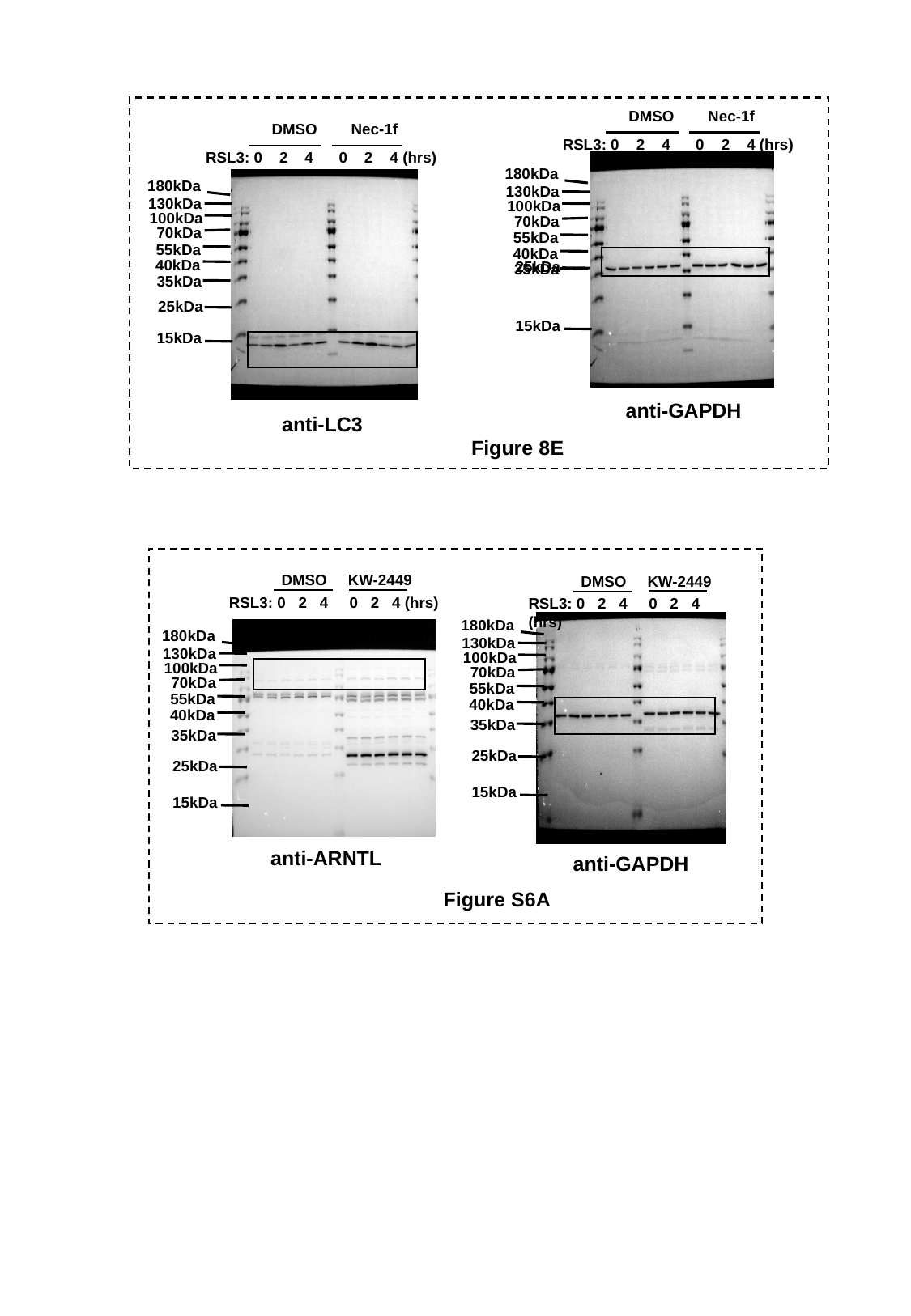

DMSO Nec-1f
DMSO Nec-1f
RSL3: 0 2 4 0 2 4 (hrs)
RSL3: 0 2 4 0 2 4 (hrs)
180kDa
180kDa
130kDa
130kDa
100kDa
100kDa
70kDa
70kDa
55kDa
55kDa
40kDa
40kDa
25kDa
35kDa
35kDa
25kDa
15kDa
15kDa
anti-GAPDH
anti-LC3
Figure 8E
DMSO KW-2449
DMSO KW-2449
RSL3: 0 2 4 0 2 4 (hrs)
RSL3: 0 2 4 0 2 4 (hrs)
180kDa
180kDa
130kDa
130kDa
100kDa
100kDa
70kDa
70kDa
55kDa
55kDa
40kDa
40kDa
35kDa
35kDa
25kDa
25kDa
15kDa
15kDa
anti-ARNTL
anti-GAPDH
Figure S6A

## Slide 8
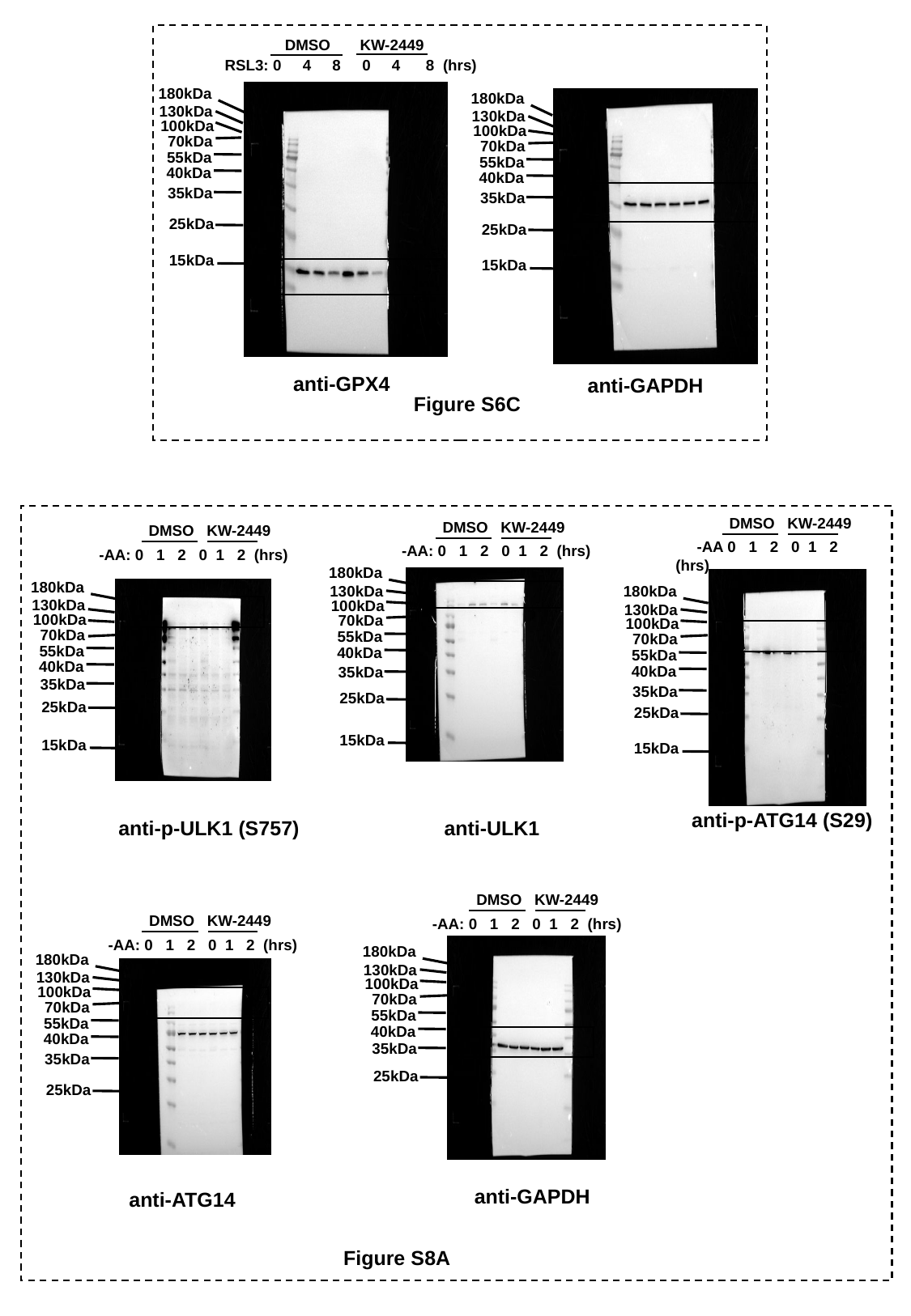

DMSO KW-2449
RSL3: 0 4 8 0 4 8 (hrs)
180kDa
180kDa
130kDa
130kDa
100kDa
100kDa
70kDa
70kDa
55kDa
55kDa
40kDa
40kDa
35kDa
35kDa
25kDa
25kDa
15kDa
15kDa
anti-GPX4
anti-GAPDH
Figure S6C
DMSO KW-2449
DMSO KW-2449
DMSO KW-2449
 -AA 0 1 2 0 1 2 (hrs)
 -AA: 0 1 2 0 1 2 (hrs)
 -AA: 0 1 2 0 1 2 (hrs)
180kDa
180kDa
130kDa
180kDa
130kDa
100kDa
130kDa
100kDa
70kDa
100kDa
70kDa
55kDa
70kDa
55kDa
40kDa
55kDa
40kDa
40kDa
35kDa
35kDa
35kDa
25kDa
25kDa
25kDa
15kDa
15kDa
15kDa
anti-p-ATG14 (S29)
anti-ULK1
anti-p-ULK1 (S757)
DMSO KW-2449
DMSO KW-2449
 -AA: 0 1 2 0 1 2 (hrs)
 -AA: 0 1 2 0 1 2 (hrs)
180kDa
180kDa
130kDa
130kDa
100kDa
100kDa
70kDa
70kDa
55kDa
55kDa
40kDa
40kDa
35kDa
35kDa
25kDa
25kDa
anti-GAPDH
anti-ATG14
Figure S8A

## Slide 9
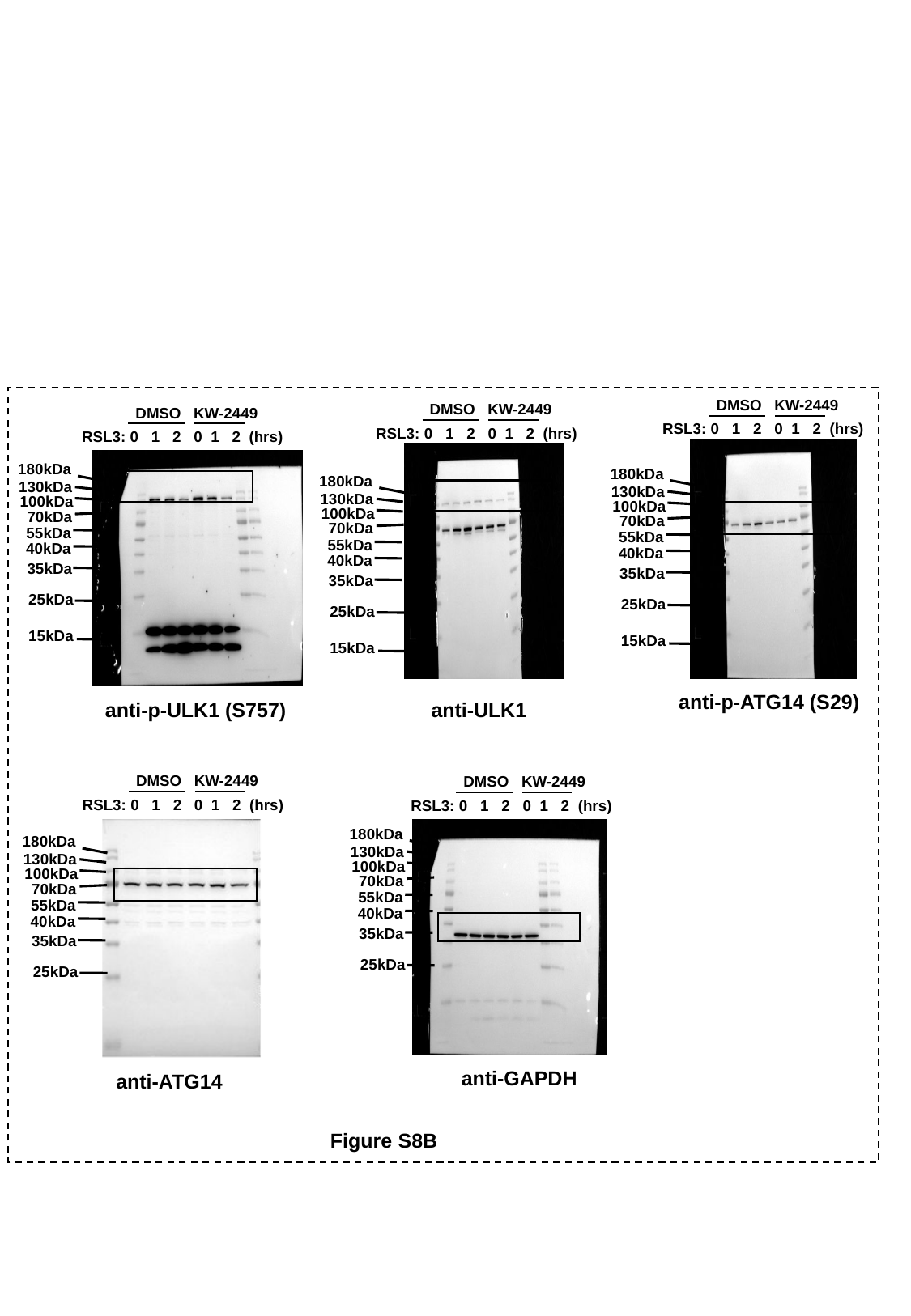

DMSO KW-2449
DMSO KW-2449
DMSO KW-2449
RSL3: 0 1 2 0 1 2 (hrs)
RSL3: 0 1 2 0 1 2 (hrs)
RSL3: 0 1 2 0 1 2 (hrs)
180kDa
180kDa
180kDa
130kDa
130kDa
130kDa
100kDa
100kDa
100kDa
70kDa
70kDa
70kDa
55kDa
55kDa
55kDa
40kDa
40kDa
40kDa
35kDa
35kDa
35kDa
25kDa
25kDa
25kDa
15kDa
15kDa
15kDa
anti-p-ATG14 (S29)
anti-ULK1
anti-p-ULK1 (S757)
DMSO KW-2449
DMSO KW-2449
RSL3: 0 1 2 0 1 2 (hrs)
RSL3: 0 1 2 0 1 2 (hrs)
180kDa
180kDa
130kDa
130kDa
100kDa
100kDa
70kDa
70kDa
55kDa
55kDa
40kDa
40kDa
35kDa
35kDa
25kDa
25kDa
anti-GAPDH
anti-ATG14
Figure S8B

## Slide 10
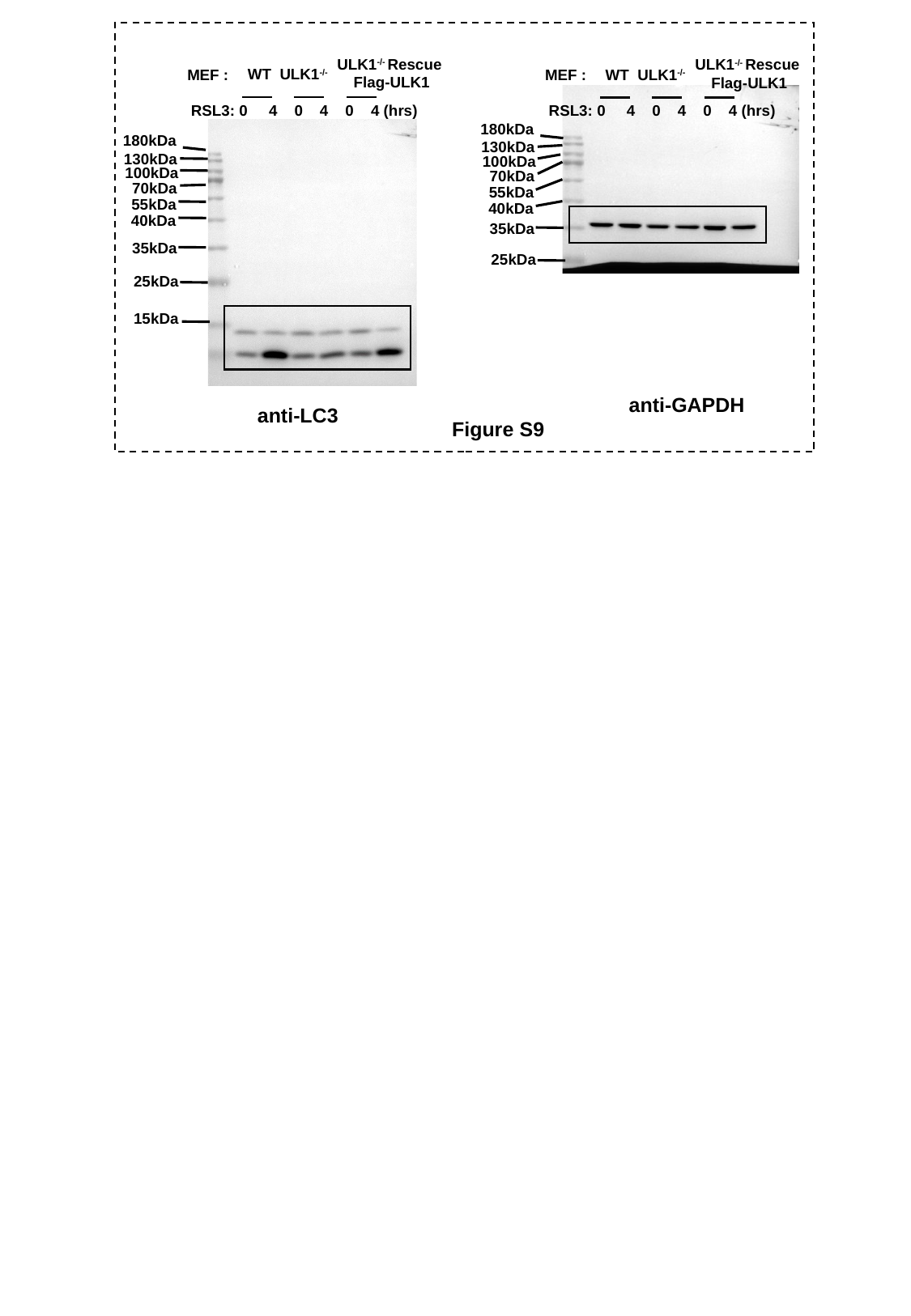

180kDa
180kDa
130kDa
130kDa
100kDa
100kDa
70kDa
70kDa
55kDa
55kDa
40kDa
40kDa
35kDa
35kDa
25kDa
25kDa
15kDa
anti-GAPDH
anti-LC3
Figure S9
ULK1-/- Rescue
Flag-ULK1
ULK1-/- Rescue
Flag-ULK1
 WT ULK1-/-
MEF :
 WT ULK1-/-
MEF :
RSL3: 0 4 0 4 0 4 (hrs)
RSL3: 0 4 0 4 0 4 (hrs)
